# A cryptic variation in a member of the Ovate Family Proteins is underlying the melon fruit shape QTL *fsqs8.1*

**DOI:** 10.1007/s00122-021-03998-6

**Published:** 2021-11-25

**Authors:** Cecilia Martínez-Martínez, Maria José Gonzalo, Pablo Sipowicz, Manuel Campos, Irene Martínez-Fernández, Carmen Leida, Mohammed Zouine, Konstantinos G. Alexiou, Jordi Garcia-Mas, María Dolores Gómez, Pablo Tornero, Miguel Ángel Pérez-Amador, Cristina Esteras, Belén Picó, Carlos Romero, Antonio J. Monforte

**Affiliations:** 1 Instituto de Biología Molecular y Celular de Plantas (IBMCP), Consejo Superior de Investigaciones Científicas (CSIC), Universitat Politècnica de València, Ingeniero Fausto Elio s/n, 46022 Valencia, Spain; 2 Instituto Nacional de Tecnología Agropecuaria (INTA), Buenos Aires, Argentina; 3 Instituto de Conservación y Mejora de la Agrodiversidad Valenciana (COMAV), Universitat Politècnica de València (UPV), Valencia, Spain; 4 Laboratoire de Recherche en Sciences Végétales, Université de Toulouse, CNRS, UPS, Toulouse INP, Auzeville-Tolosane, France; 5 Centre for Research in Agricultural Genomics (CRAG), CSIC-IRTA-UAB-UB, Edifici CRAG, Campus UAB, Bellaterra, Barcelona, Spain; 6 Institut de Recerca i Tecnologia Agroalimentàries (IRTA), Bellaterra, Barcelona, Spain; 7 Present Address: Universidad de Almería, Almería, Spain

## Abstract

**Key message:**

The gene underlying the melon fruit shape QTL *fsqs8.1* is a member of the Ovate Family Proteins. Variation in fruit morphology is caused by changes in gene expression likely due to a cryptic structural variation in this locus.

**Abstract:**

Melon cultivars have a wide range of fruit morphologies. Quantitative trait loci (QTL) have been identified underlying such diversity. This research focuses on the fruit shape QTL *fsqs8.1*, previously detected in a cross between the accession PI 124112 (CALC, producing elongated fruit) and the cultivar ‘Piel de Sapo’ (PS, producing oval fruit). The CALC *fsqs8.1* allele induced round fruit shape, being responsible for the transgressive segregation for this trait observed in that population. In fact, the introgression line CALC8-1, carrying the *fsqs8.1* locus from CALC into the PS genetic background, produced perfect round fruit. Following a map-based cloning approach, we found that the gene underlying *fsqs8.1* is a member of the Ovate Family Proteins (OFP), *CmOFP13,* likely a homologue of *AtOFP1* and *SlOFP20* from *Arabidopsis thaliana* and tomato, respectively. The induction of the round shape was due to the higher expression of the CALC allele at the early ovary development stage. The *fsqs8.1* locus showed an important structural variation, being *CmOFP13* surrounded by two deletions in the CALC genome. The deletions are present at very low frequency in melon germplasm. Deletions and single nucleotide polymorphisms in the *fsqs8.1* locus could not be not associated with variation in fruit shape among different melon accessions, what indicates that other genetic factors should be involved to induce the CALC *fsqs8.1* allele effects. Therefore, *fsqs8.1* is an example of a cryptic variation that alters gene expression, likely due to structural variation, resulting in phenotypic changes in melon fruit morphology.

**Supplementary Information:**

The online version contains supplementary material available at 10.1007/s00122-021-03998-6.

## Introduction

Melon (*Cucumis melo* L.) is a crop species that belongs to the Cucurbitaceae family, which also includes important cultivated species such as cucumber (*Cucumis sativus* L.), watermelon (*Citrullus lanatus* Thumb.), pumpkins and squashes (*Cucurbita* spp.), gourds [*Momordica charantia* L., *Lagenaria siceraria* Molina. Standley, *Benincasa hispida* (Thunb.) Cogn., *Luffa aegyptiaca* Mill. *Luffa acutangula* L. Roxb., *Trichosanthes cucumerina* L.] and other minor crops as *Cucumis metuliferus* Meyer ex Naudin *and Cucumis anguria* L. Together with watermelon and cucumber, melon is one of the cucurbit crops with the greatest economic impact. Worldwide production has increased since the beginning of the 21st Century from 20 million to more than 27 million tons (FAOSTAT 2021).

Melon was domesticated independently at least twice in Africa and Asia (Endl et al. 2018; Zhao et al. 2019), although the cultivars that are commonly grown worldwide derived from the Asian domestication through divergent diversification from the Indian subcontinent to Oriental and Occidental Eurasian regions (Gonzalo et al. 2019; Zhao et al. 2019). One of the consequences of this diversification was the development of a large number of landraces, traditional and modern cultivars that show an impressive diversity in fruit morphology, from the small (30–50 g) round fruit of wild melons to the large and extremely elongated fruit of *flexuosus* varieties (Monforte et al. 2014). Pitrat (2017) proposed an exhaustive horticultural classification of melon that reflects such phenotypic diversity, defining nineteen groups, one wild (agrestis), two semi‐domesticated or feral (kachri and chito) and sixteen domesticated and cultivated ones.

The genetic control of melon fruit morphology has been studied in several works, and quantitative trait loci (QTL) and consensus QTLs have been defined (reviewed by Monforte et al. 2014; Pan et al. 2020). The pleiotropic effects of two genes, *a* and *p*, on fruit shape are widely known (Perin et al. 2002): *a* gene controlling female flower sex expression, being strictly female or hermaphrodite in monoecious or andromonoecious varieties, respectively, whereas *p* controls the fruit carpel number (with three carpels being common for most varieties and five for a few other). Regarding the pleiotropic effects observed for *a*, fruit from strictly female flowers (in monoecious plants) are usually much more elongated than fruit from hermaphrodite flowers in andromonoecious plants (Monforte et al. 2005; Abdelmohsin and Pitrat 2008). Gene *a* encodes a *1-AMINOCYCLOPROPANE-1-CARBOXYLIC ACID SYNTHASE* (*CmACS-7*), and a missense mutation in the gene produces a loss of CmACS-7 protein activity, leading to the development of stamens in the female flower (andromonoecy), which results in subsequent pleiotropic effects making a rounder fruit shape (Boualem et al. 2008). Whereas wild melons are monoecious, the andromonoecious mutation was selected during cultivar diversification. Traditional Indian landraces (the primary center of melon diversity) are monoecious, while andromonoecy is more common in Mediterranean and modern cultivars. Both andromonoecious and monoecious cultivars need pollinators to ensure proper fruit production, so the selection of the andromonoecious mutation does not seem to be related to an increase in fruit set. It is likely that the selection sought rounder fruit. On the contrary, the increase of in the fruit carpel number, controlled by *p*, has been associated with rounder fruits in several mapping populations (Perin et al. 2002; Monforte et al. 2004). Wild melons and most traditional and modern cultivars develop three carpels, while five carpels are found only in a small subset of Indian and Oriental cultivars. Strong evidences based on investigations performed in cucumber (Li et al. 2016), co-localization of candidate genes with the *p* locus in experimental mapping populations (Pan et al. 2020) and a genome-wide association study (GWAS) panel indicate that the gene *p* may be the melon homologue (*CmCLV3*) of *CLAVATA3* which tomato homologue (*SlCLV3*) controls locule number in the tomato fruit (Xu et al. 2015). For the rest of the QTLs, candidate genes belonging to the *CELL NUMBER REGULATOR* (*CNR/FW2.2)*, *SlKLUH/CYP785A/FW3.2*, *OVATE*, *SUN, TON1 RECRUTING MOTIF (TRM)*, *CELL SIZE REGULATOR* (*CSR/FW11.2*)*, YABBY*, and *WOX* families have been proposed based on the co-location of members from these families with melon fruit morphology QTLs (Monforte et al. 2014; Pan et al. 2020). Among them, *OVATE, CSR* and *SUN* showed a larger number of co-locations (Pan et al. 2020), although no functional verification has been carried out until now.

Diaz et al. (2014) mapped several fruit shape QTLs in a segregating population derived from the cross between the Indian landrace PI 124,112 (CALC, producing elongated fruit) and the Spanish “Piel de Sapo” (PS, producing oval fruit) cultivar. The QTL with the strongest effect was mapped to chromosome 8 and was named *fsqs8.1*. Surprisingly, round fruits were induced by the CALC allele, which explained the transgressive segregation observed for fruit shape in this population. The QTL was introgressed into PS by developing the introgression line (IL) CALC8-1, which carries a segment of chromosome 8 from CALC containing the *fsqs8.1* locus, while the rest of the genome was PS. CALC8-1 produced round fruits, verifying the effect of *fsqs8.1* (Diaz et al. 2014). Interestingly, *fsqs8.1* maps to the same genome region as the consensus QTL *FSQM8* (Monforte et al. 2014) and *CmFSI8.3* (Pan et al. 2020). Additionally, Gur et al. (2017) showed a strong association of fruit shape in the same chromosome 8 genomic region in a diverse melon germplasm collection by GWAS. These works indicated that *fsqs8.1* could be one of the major loci responsible for the enormous fruit shape variability observed in melon germplasm. Wu et al. (2018) fine-mapped *fsqs8.1* between markers Sca76-197 and Sca76-45 on chromosome 8. Several genes were identified in that region, including a Serine/threonine kinase (MELOC025211), a 1-aminocyclopropane-1-carboxylate oxidase (MELO3C025210) and a member of the OFP family (MELO3C025206) that was previously named as *CmOFP13* by Monforte et al. (2014). Among them, *CmOFP13* was pointed out as the strongest candidate gene.

The objective of the present work is to identify the causal gene underlying *fsqs8.1*, to track the history of its variability during melon diversification, to shed light on its effects during ovary development, and to investigate its genetic effects in several genetic landscapes.

## Material and methods

### Plant material

Diaz et al. (2014) previously developed the introgression line CALC8-1 that carries an introgression on chromosome 8, covering the *fsqs8.1* locus, from the traditional melon accession PI 124,112 (CALC) from Bihar, India, into the “Piel de Sapo” (PS) cultivar from Spain (Oliver et al. 2001). Eduardo et al. (2005) developed the introgression line SC8-3, which carries an introgression with *fsqs8.*1 locus from the Korean cultivar “Songwan Charmi” (SC) accession PI161375 in the same PS genetic background. The three genotypes are andromonoecious. Large F_2_ populations were developed from crosses between CALC8-1 and PS for fine mapping. SC8-3 and CALC8-1 were also crossed to study gene interaction between alleles.

An array of melon cultivars and accessions from different world regions and belonging to several melon horticultural groups was obtained from the Instituto de Conservación y Mejora de la Agrodiversidad Valenciana (COMAV, Polytechnic University of Valencia, Spain) germplasm bank (Supplementary Table S1) to study associations between Single Nucleotide Polymorphism (SNP) markers tightly linked to *fsqs8.1* and fruit shape. A set of five of them were selected and crossed with CALC8-1 and PS to investigate interaction between *fsqs8.1* and the genetic background. The cultivar ‘Mucha Nesvi’ (MN), a monoecious ameri cultivar, was crossed with CALC8-1 to verify the epistatic interaction for fruit shape between the sex expression gene *a* and *fsqs8.1*.

### Positional cloning

Three thousand five hundred F_2_ seedlings from the CALC8-1 × PS cross were screened for the markers flanking the *fsqs8.1* position Sca76-197 and Sca76-42 and the internal marker Sca76-140b (Supplementary Table S2). DNA was extracted according to Doyle and Doyle (1990) with minor modifications. Genotyping was carried out with the KASPAR technology (Smith and Maughan 2015) by the Genotyping Service at the John Innes Center (Norwich, UK). Recombinants were transferred to the greenhouse to obtain self-seed. The position of the recombinant events was established by genotyping the recombinant plants with an array of SNP markers (Supplementary Table S2) distributed along the *fsqs8.1* locus by KASPAR and High Resolution Melting (HRM, Wittwer et al. 2003) with MELTDOCTOR™ (ThermoFisher Scientific, Waltham, MA) following the supplier recommendations. Seedlings from selected F_3_ families were genotyped with the appropriate markers to identify homozygous seedlings for CALC and PS for the corresponding segregating recombinant introgression. The selected seedlings were transferred to greenhouse. Plants from each family were planted in the same row in the greenhouse, randomly distributed within each family. The fruit shape index (FSI) was defined as the ratio between maximum fruit length and maximum fruit diameter. The mean FSI for the groups of plants with a contrasting allele composition at the segregating introgression within each F_3_ family were compared with a *t-*test to determine if *fsqs8.1* was present or not in the respective introgressions.

### Sequencing and genomic analysis of *fsqs8.1* locus

Amino acid sequences of tomato, *Arabidopsis thaliana* and melon OVATE family protein (OFP) members, were retrieved from previous works (Monforte et al. 2014) and aligned with CLUSTAL. Evolutionary distances were computed using the Poisson correction method (Zuckerkandl and Pauling 1965) in units of the number of amino acid substitutions per site, removing ambiguous positions for each sequence pair. The tree was calculated with the neighbor-joining method (Saitou and Nei 1987), and the statistical support of the clusters was assessed by a bootstrap test with 2000 replicates (Felsenstein et al. 1985). All the analysis were conducted with MEGA X (Kumar et al. 2018).

MELO3C025206 was amplified from PS, CALC8-1 and SC8-3 using the Cmofp13 primers (Supplementary Table S2) with *Taq I* DNA polymerase (Biotools, Madrid, Spain). The PCR products were cleaned with ExoSap-IT™ (ThremoFisher Scientific) and sequenced by the Sequencing Service at the Instituto de Biología Molecular y Celular de Plantas (IBMCP; Valencia, Spain) with an ABI3130 Genetic analyzer (Applied Biosystems, Foster City, CA). The sequences were aligned with CLUSTAL implemented in MEGA X (Kumar et al. 2018).

Illumina raw sequences from melon accessions (Zhao et al. 2019) were retrieved from the Sequence Read Archive (SRA) under the NCBI BioProject with the accession ID PRJNA565104. Adaptors and low-quality regions were removed from the reads using cutadapt (Martin 2011). Clean, high-quality reads were then mapped to the melon reference genome v4.0 (Castanera et al. 2020) using BWA-MEM (Li 2013), and the mapping results were visualized with the Integrative Genomics Viewer IGV (Robinson et al. 2011).

The 10 × Chromium sequencing was performed at the GeT-PlaGe core facility, INRA Toulouse (France). The Chromium libraries were prepared according to 10X Genomics’ protocols using the Genome Reagent Kits v2. The samples quantity and quality controls were validated on Qubit, Nanodrop and Femto. The optimal performance was characterized on input gDNA with a mean length greater than 50 kb. The libraries were prepared from 3 µg of HMW gDNA (cut off at 50 Kb using BluePippin system) or 0.625–1.8 ng of HMW DNA. Briefly, in the microfluidic Genome Chip, a library of Genome Gel Beads was combined with HMW template gDNA in Master Mix and partitioning oil to create Gel Bead-In-EMulsions (GEMs) in the Chromium. Each gel bead was functionalized with millions of copies of a 10x™ Barcoded primer. Upon dissolution of the genome gel bead in the GEM, primers containing (i) an Illumina R1 sequence (Read 1 sequencing primer), (ii) a 16 bp 10 × Barcode, and (iii) a 6 bp random primer sequence were released. A read 1 sequence and the 10x™ Barcode were added to the molecules during GEM incubation. P5 and P7 primers, Read 2, and Sample Index were added during library construction. 10 cycles of PCR were applied to amplify libraries. Library quality was assessed using a fragment analyzer, and libraries were quantified by q-PCR using the Kapa Library Quantification Kit. The libraries were performed on an Illumina HiSeq3000 using a paired-end read length of 2 × 150 pb with the Illumina HiSeq3000 sequencing kits. The 10 × Genomics chromium libraries were assembled using the 10 × Genomics Supernova software (Weisenfeld et al. 2017), using default parameters.

### Ovary development analysis and RT-PCR

For the development study, ovaries from the three genotypes CALC8-1, SC8-3 and PS were collected fresh at different stages: E1 (carpel primordial initiated), E2 (primordial stigmas appear), E3 (stigma and style defined and anthers with pollen) and E4 (ovule primordial initiated) that correspond to stages 6, 8-1, 8-2, 8-3, respectively, from Bai et al. (2004). The ovaries were fixed overnight in 4% (w/v) p-formaldehyde in 0.1 M sodium phosphate pH 7.2 with 0.05% (v/v) of Tween 20 at 4 °C and dehydrated in a graded ethanol series as described in Gomez et al. (2004, 2011). After dehydration, the samples were embedded in Paraffin wax (Paraplast Plus). To obtain the ovary sections, samples in paraffin were sectioned in a Leica RM2025 microtome. The sections were stained with 0.02 toluidine blue, and the images of ovaries were captured using a Leica DM5000 microscope equipped with the LAS X application at the microscopy services at the IBMCP.

Additionally, flowers from six days prior anthesis to full anthesis stages were collected freshly and sectioned longitudinally. The ovary hair was eliminated to facilitate the definition of ovary contour. Ovaries were observed with a Leica DMS 1000 stereomicroscopy equipped with the LAS X application for image capture at the microscopy service of the IBMCP.

Gene expression analysis during flower development was performed in the previously defined four development stages in PS, SC8-3 and CALC8-1. Five independent replicates were analyzed. RNA extractions were performed according to the protocol from the Aurum Total RNA Mini kit (Biorad, Hercules, CA)) and treated with Ambion DNAse I (RNAse-free) to eliminate DNA. cDNA was then synthesized from 1 µg of total RNA using PrimeScript 1st strand cDNA synthesis kit. The expression of genes was evaluated through quantitative RT-PCR (q-PCR) by using the TAKARA SBRY Premix ExTaq (Tli RNAseH Plus). Supplementary Table S2 shows the corresponding primers used for q-PCR. The q-PCR primers were designed from the coding regions of each gene by using the *Primer3* software (Untergasser et al. 2012). To avoid possible cross-amplification, and before any q-PCR experiment, the homology of each primer was checked using the BLAST tool from MELONOMICS against the CM4.0 genome (www.melonomics.net) and the expected size tested in agarose gels. The q-PCR reactions consisted of two step of hold at 95 °C 30 s and 40 cycles of 3 s at 95 °C, and 30 s at 60 °C. The relative expression of each gene was determined by the comparative Ct (*Cycle Threshold*) method using *C. melo ACTIN* gene (MELO3C023264) as the internal standard. The efficiency of amplification for each amplicon was tested using increasing amounts of template cDNA, and were all roughly equivalent. The absolute value of the ΔCt slope (Ct of the target gene-Ct of the reference gene) versus serial dilutions of cDNA for a given sample must be less than 0.1. The relative expression of each gene was then calculated relative to a calibrator sample using the formula 2^−ΔΔCt^, where ΔΔCt is the difference between the ΔCt of each sample and the ΔCt of the calibrator sample. Means of relative expression between genotypes within each ovary development stage were compared with the nonparametric Kruskal–Wallis test.

### *Arabidopsis thaliana* transformation

MELO3C025206 open reading frame from both CALC8-1 was inserted into pDONR222 (Invitrogen, Waltham, MA) by PCR with two specific oligonucleotides (GGGGACAAGTTTGTACAAAAAAGCAGGCTTCGAAGGAGATAGAACCATGAGAAATCACAAGTTCCGT and GGGGACCACTTTGTACAAGAAAGCTGGGTTTCAAAGAGGTGGAGGAGAAGG) using a BP Gateway reaction (Invitrogen). Upon confirmation of the sequence, cDNA was transferred to pGWB14 (Nakagawa et al. 2007) to render MPA635 (35S:OVATE_CAL8-1), and it was introduced in *Arabidopsis thaliana* (L.) Heynh. (ecotype Col-0) by *Agrobacterium*-mediated floral-dip transformation (Clough and Bent 1998). T1s, T2s, and T3s lines were selected by the presence of hygromycin (Duchefa Biochemie, Haarlem, The Netherlands) in Murashige and Skoog (MS) medium plates (Murashige and Skoog 1962) (Duchefa). Several T2 lines were inspected, and four representative lines were fixed for the transgene in homozygosis. Seeds were sterilized in ethanol and germinated in MS for 4 days at 4 °C in the dark, followed by 7–8 days at 22 °C under a long day photoperiod (16 h/8 h). Seedlings were then transferred to soil and grown in a chamber at 22 °C/19 °C under long day photoperiod (16 h/8 h). Pictures from seedlings at day 9, flowers, pistils, and fruits were taken with a SMZ1270 stereomicroscope (Nikon, Leuven, Belgium), whereas those from the rosettes of adult plants were obtained with an Alpha ILCE-6000L digital camera (Sony, Tokyo, Japan).

### Association of *fsqs8.1* locus haplotypes with fruit shape in melon germplasm

Sixty-six melon accessions (Supplementary Table S1) were grown in COMAV greenhouse facilities in 2014 and 2018 seasons in a completely randomized design with three biological replicates and the FSI was obtained for each one. Accessions were genotyped with SNPs Sca76-snp192, Sca76-snp155, Sca76-snp140 and the sca76-95indel-ov makers (Supplementary Table S2) located in the *fsqs8.1* locus. Associations between the markers and the FSI were studied by ANOVA. Haplotypes were defined via visual inspection. FSI means of the haplotypes with at least five accessions were compared with Tukey’s multiple comparison test (*p* < 0.05). Statistical analyses were carried out with JMP® v. 11 (SAS Institute Inc. Cary, NC).

### Interactions of *fsqs8.1* in different genetic situations

To study the gene action between the CALC and SC *fsqs8.1* alleles five plants of each, CALC8-1, SC8-3 and the hybrid CALC8-1-x-SC8-3 were grown in a completely randomized design in the COMAV greenhouse facilities. Fruits were collected at full mature stages and the FSI index recorded. The gene action (d/[a]), with d being the dominance deviation and [a] the absolute additive value) was calculated as:

d/[a] = [ CALC8-1-x-SC8-3-(CALC8-1-SC8-3)/2]/[(CALC8-1 + SC8-3)/2].

with the contrast 2*CALC8-1-x-SC8-3-(CALC8-1-SC8-3) = 0 to determine the statistical significance of the gene action.

Regarding the interaction of the *fsqs8.1* alleles with the *a* sex expression gene, two hundred seedlings belonging to the F_2_ population derived from the cross CALC8-1-x- ‘Mucha Nesvi’ (MN) were genotyped using *fsqs8.1* SNPs markers and the perfect marker for the *a* gene ALU-CAPS (Boualem et al. 2008) to select plants homozygous for the four possible allelic combinations at both genes. Plants were grown in the COMAV greenhouse facilities using a completely randomized design. The interaction between genes was contrasted by a two-way ANOVA.

Finally, interaction of *fsqs8.1* alleles with different genetic backgrounds (GB) was performed in F1 hybrids from the crosses between CALC8-1 and PS with an array of six different melon cultivars (Supplementary Table S1), having, therefore, the CALC or PS *fsqs8.1* alleles in heterozygous conditions. The hybrids were grown in two consecutive years (2016 and 2017) at the COMAV greenhouse facilities in a completely randomized design with ten replicated. The effect of the genetic background, *fsqs8.1* allele, year and their respective interactions were assessed by the ANOVA model:

$${Y}_{{{\text{ijkl}}}} = \, \mu \, + FSQS8.1_{i} + {\text{GB}}_{{\text{j}}} + {\text{ Year}}_{{\text{k}}} + FSQS8.1*{\text{GB}}_{{{\text{ij}}}} + FSQS8.{1}*{\text{Year}}_{{{\text{ik}}}} + {\text{GB}}*{\text{Year}}_{{{\text{jk}}}} + {\text{ e}}_{{{\text{ijkl}}}}$$

where *FSQS8.1*_i_ indicates the CALC or PS *fsqs8.1* allele in the hybrid in heterozygous conditions,GB_j_ the effect of the different backgrounds, Year_k_ the effect of the year, and the respective two-way interactions. Statistical analyses were carried out with JMP® v. 11 (SAS Institute Inc. Cary, NC).

## Results

### Fine mapping of *fsqs8.1*

**Fig. 1:**
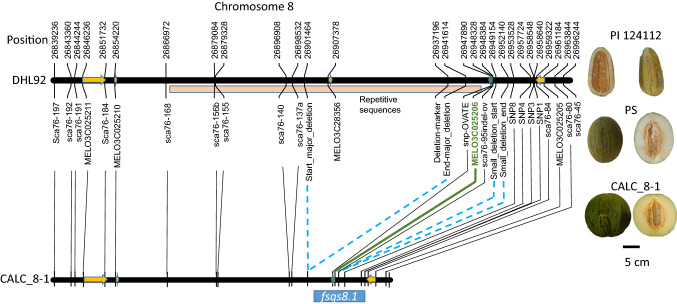
High-resolution mapping of *fsqs8.1*. A schematic representation of the reference genome V4.0 (Castanera et al. 2020) based on the double haploid line DHL92 is depicted in the upper part of the figure, indicating the position of markers, annotated genes and the repetitive sequence rich region. The length of the genes and the direction of the transcription are indicated with arrows. The deduced genome of CALC8-1 based on re-sequencing data is depicted in the lower part. Blue dashed lines indicates the large deletions observed in CALC8-1. The position of *fsqs8.1* based on the fine mapping experiments is indicated below the CALC8-1 genome. The candidate MELO3C025206 is highlighted in green. On the right, typical fruits of PI 124,112 (CALC, donor parent), PS (recipient parent) and CALC8-1 (introgression line carrying the *fsqs8.1* locus from CALC in PS background) are also shown) (color figure online)

The genomic position of *fsqs8.1* was previously defined between markers Sca76-197 and Sca76-45 on chromosome 8 of the melon genome (Wu et al. 2018). According to the CM4.0 melon genome version (Castanera et al. 2020), the physical distance of those markers is 169 kb (Fig. 1) between positions 26,839,235 and 27,008,844, which carries five annotated genes: MELO3C025211 (serine/threonine kinase), MELO3C025210 (gibberellin 2-beta-dioxygenase 2-like), MELO3C028356 (hypothetical unknown protein with some homology to a retrotransposon gag protein), MELO3C025206 (ovate family protein), and MELO3C025205 (RING-type E3 ubiquitin transferase). Interestingly, the 93 kb region between MELO3C025210 and MELO3C025206 is composed by long terminal repeats (LTR) and repetitive elements, characteristics of retrotransposons, and only contains one annotated gene, MELO3C028356.

A fine mapping strategy was followed in order to reduce the number of candidate genes that might underlie *fsqs8.1*. Sequence reads previously obtained by Sanseverino et al. (2015) from PS, CALC, and SC accessions were mapped to the melon reference genome CM4.0 to identify SNPs for the subsequent fine mapping efforts. The reference genome was obtained from the double haploid line DHL92 derived from the cross between PS and SC. The genomic region including the *fsqs8.*1 locus in DHL92 corresponds to the PS genome. Twenty-five variant calls were identified; however, no variant calls were found in the 49 kb region between the positions from 26,898,531 to 26,947,890, corresponding to the previously mentioned LTR-rich region (Fig. 1). A visual inspection of PS, SC and CALC read mappings with IGV (Robinson et al. 2011) showed that no CALC Illumina reads mapped to this genomic region, except for some low-quality read mappings corresponding to repetitive sequences, while mapping reads were observed for PS and SC (Supplementary Fig. S1). Furthermore, a second smaller region lacking CALC Illumina reads at 5' of MELO3C025206 was also observed. In order to verify that this pattern was also present in the IL CALC8-1 genome, the IL and PS were re-sequenced using 10X Chromium technology. Although the assembly in this genomic region was not of sufficient quality, the read mapping pattern for both genotypes was similar as the one observed in the previous re-sequencings, confirming the presence of two deletions surrounding MELO3C025206 (Supplementary Fig. S1) in the CALC8-1 genome. A PCR marker was developed to amplify a sequence within the putative large deletion; the deletion-marker failed to amplify either CALC8-1 or CALC but produced a clear amplification product in PS (Supplementary Fig. S1). These results suggested a large 40 kb deletion, approximately from positions 26,900,000 to 26,938,000, and an smaller 3 kb deletion from positions 26,949,154 to 26,952,139 (Fig. 1) in the CALC *fsqs8.1* locus.

We obtained seven highly informative recombinants in the *fsqs8.1* locus after two rounds of recombination screening of 3500 F_2_ CALC8-1xPS seedlings, with recombination points among the annotated genes (Supplementary Table S3). The progeny analysis of those recombinants showed the *fsqs8.1* locus expanded from positions 26,937,197 to 26,958,640. The only coding sequence in that region corresponded to *MELO3C025206* (Fig. 1), strongly suggesting that this gene is underlying the *fsqs8.1* QTL. BLAST analysis showed that *MELO3C025206* encodes for a member of the ovate family proteins, previously named by Monforte et al. (2014) as *CmOFP13*. The direction of the translation was reverse to the reference genome, i. e., the sequence up to 26,937,197 was downstream to MELO3C025206, and sequence up to 26,958,640 upstream.

### Ovary development and gene expression

**Fig. 2:**
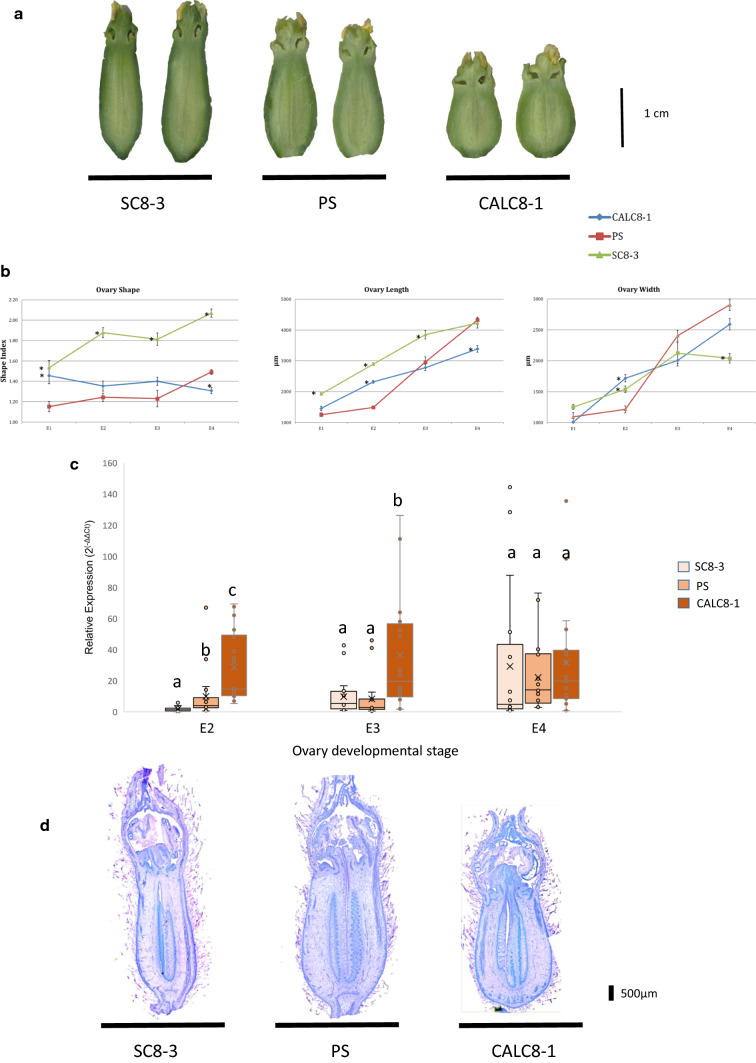
Developmental analysis and q-PCR of SC8-3, PS and CALC8-1 ovaries. **a** Ovaries from the three genotypes at anthesis. **b** Ovary length, width (both in µm) and shape index for the three genotypes through ovary development stages: E1 (carpel primordial initiated), E2 (primordial stigmas appear), E3 (stigma and style defined and anthers with pollen) and E4 (ovule primordial initiated). Asterisks (*) indicates means of CALC8-1 or SC8-3 ovaries statistically significant different (*p* < 0.05) from PS means **c** Accumulation of MELOC025206 transcripts estimated by qRT-PCR between genotypes and developmental stages. Different letters indicate statistically significant differences among genotypes within developmental stages (*p* < 0.05). **d** Paraffin sections of representative ovaries from the three genotypes at E4

The final melon fruit shape is usually determined pre-anthesis during ovary development (Perin et al. 2002; Eduardo et al. 2005). Ovary shape was studied in three genotypes: PS, CALC8-1 and SC8-3. SC8-3 is an IL that carries an introgression in the *fsqs8.1* locus from SC (Eduardo et al. 2005). As fruits from SC8-3 are very elongated (Eduardo et al. 2007), it is likely that the three genotypes represent an allelic series for *fsqs8.1* ranging from elongated (SC8-3), oval (PS) to round (CALC8-1). Differences in ovary shape at anthesis and pre-anthesis were observed among them (Fig. 2a, Supplementary Fig. S2), CALC8-1 showed a clear rounder shape four days before anthesis (DBA) and SC8-3 an elongated shape even at 6 DBA. To find the ovary developmental stage when the differences in ovary shape appeared, ovaries were collected in four early developmental stages: E1 (carpel primordial initiated), E2 (primordial stigmas appear), E3 (stigma and style defined and anthers with pollen) and E4 (ovule primordial initiated). Differences in ovary shape were already evident for SC8-3 at E2, with elongated ovaries compared to PS and CALC8-1, whereas CALC8-1 showed rounded ovaries compared to PS at E4 (Fig. 2b). Regarding ovary length and width, PS showed strong growth increase between E2 and E3 compared with CALC8-1, suggesting that there was already an inhibition of the growth in CALC8-1 at this stage that ended up with the variation in shape observed at E4 stage.

Next, we wanted to correlate the expression of *CmOFP13* with the variation of ovary shape through the ovary developmental stages among PS, SC8-3 and CALC8-1. Expression of *CmOFP13* between the E2 and E4 stages showed different patterns among genotypes (Fig. 2c). *CmOFP13* was residually expressed in SC8-3 at E2 and low levels at E3, whereas in PS the expression was low (although higher than SC8-3 in E2) in those stages, but, on the contrary, the expression was high in CALC8-1 in those stages. Finally, the expression was similar among genotypes in E4. Therefore, the correlation between *CmOFP13* and ovary shape was observed: lack of expression in E2 for elongated shape, low expression in E2 and E3 with oval shape and high expression in E2 and E3 with round shape. Figure 2d depicts representative ovaries at E4, where the three different ovary shapes were evident among all three genotypes.

### Characterization of *CmOFP13* gene sequence and surrounding genomic region

**Fig. 3:**
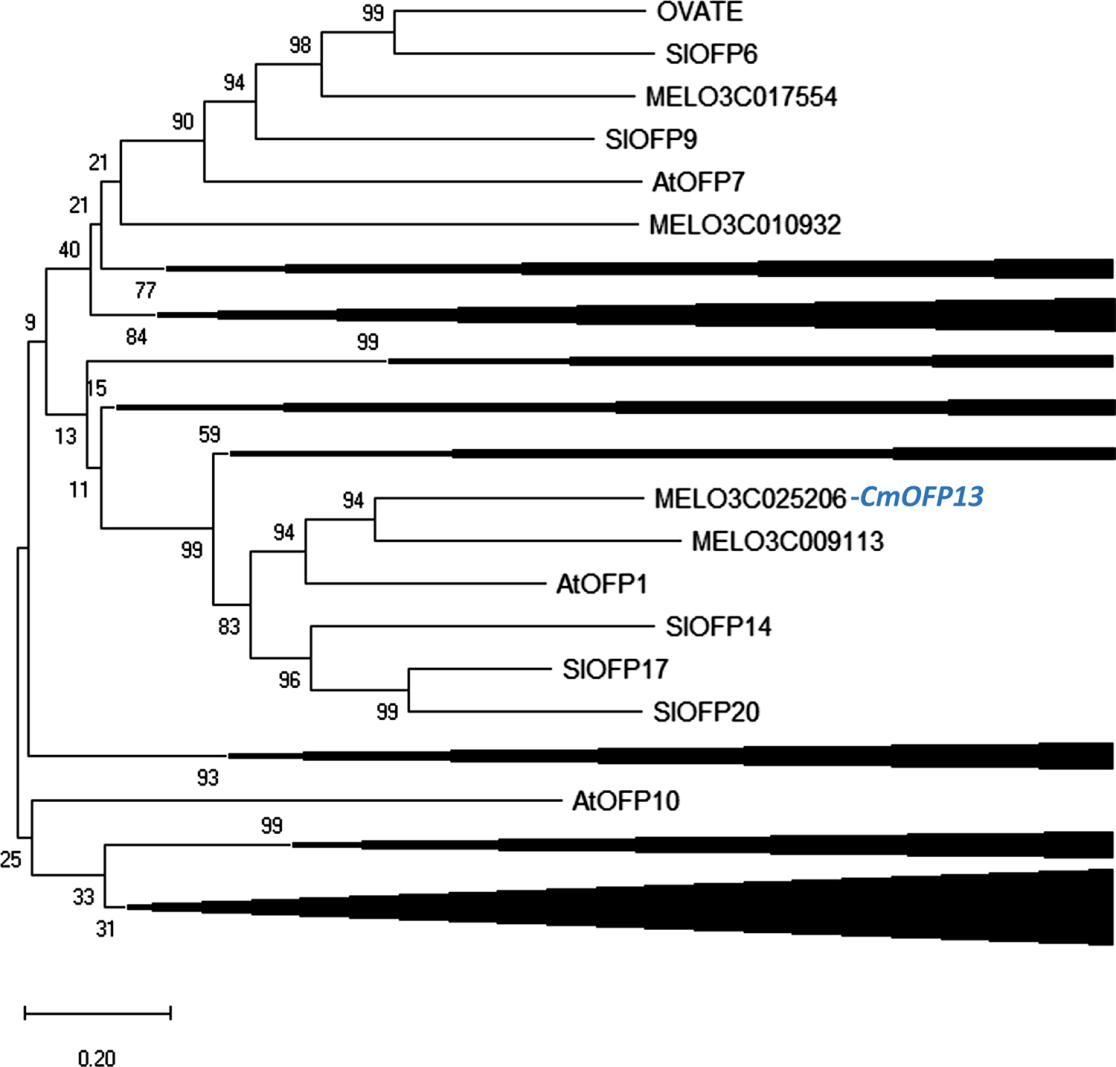
Phylogeny of *Arabidopsis thaliana*, tomato and melon ovate family protein genes based on Poisson distances (Zuckerkandl and Pauling 1965). Bootstrap values were based on 2000 replicates. Evolutionary analyses were conducted in MEGA X (Kumar et al. 2018). In order to simplify the figure, the nodes that do not include informative members for the current report are collapsed. The full phylogenetic tree is depicted in Fig. S2

*CmOFP13* has a 999-nucleotide open reading frame, encoding for a protein compromised of 331 amino acids with no introns. CmOFP13 includes an ovate sequence motif, therefore likely being a member of the OVATE family proteins (OFP). This family has been extensively studied especially in *A. thaliana* (Wang et al. 2016), tomato (Rodriguez et al. 2011) and other plant species (Dangwal and Das 2018). Monforte et al. (2014) identified the melon gene members belonging to this family. A phylogenetic analysis was carried out with *A. thaliana*, tomato and melon OFPs (Fig. 3, Supplementary Fig. S3). *CmOFP13* clustered with *AtOFP1* and *SlOFP20*, and it is likely a homolog of these genes. More importantly, *AtOFP1* and *SlOFP20* induce organ shortening in their respective species (Hackbusch et al. 2005; Wu et al. 2018). Therefore, phylogenetic analysis reinforced the putative role of *CmOFP13* in the regulation of organ shape in melon.

*CmOFP13* coding sequences from SC8-3, CALC8-1 and PS *CmOFP13* were obtained with Sanger technology. Only one synonymous mutation was found between PS and CALC8-1. The same mutation was also present in SC8-3 plus two additional synonymous mutations (Supplementary Fig. S4). On the other hand, sequence variations were observed in the surrounding upstream and downstream non-coding regions (Supplementary Figure S5). In the downstream sequence, 24 SNPs were found in PI161375 and the deletion of approximately 40 kb at 3 kb from the stop codon of *CmOFP13* coding sequence in PI124112 compared with the reference sequence (PS). In the case of upstream sequence, 123 and 114 SNPs were found in PI161375 and PI124112, respectively. The 3-kb deletion in PI124112 is located at −724 nucleotides from the transcription start point (according the annotation of accession XP_016903000 in www.ncbi.nlm.nih.gov). Therefore, these variations may affect the promoter sequences of *CmOFP13* and most probably the expression profile of the gene but not preventing it completely*.* Thus, lack of non-synonymous mutations in the coding regions, the existing sequence variability in the surrounding regions and the differences of gene expression between PS and CALC8-1 strongly suggested that the changes in organ shape induced by *CmOFP13* in CALC8-1 might be due to changes in gene expression, not differences in protein function between alleles.

**Fig. 4:**
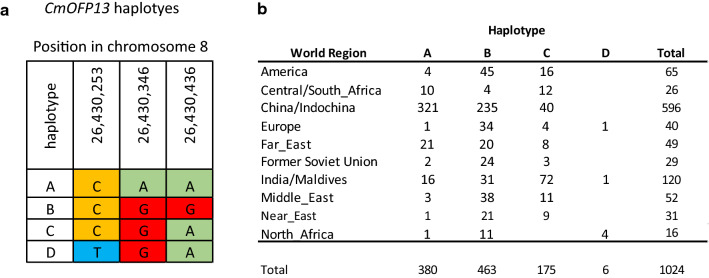
Haplotypes found for *CmOFP13* among 1175 re-sequenced melon accessions (Zhao et al. 2019). **a** SNPs defining the four haplotypes and their position on the melon genome v4.0 (Castanera et al. 2020). CALC8-1 shares the same haplotype as PI 124,112 **(c)**, while PS shows the B haplotype. **b **Distribution of the four haplotypes through accessions belonging to different world regions

The sequence variation in *CMOFP13* across melon germplasm was studied in 1175 re-sequenced accessions (Zhao et al. 2019). Three synonymous mutations were identified in the *CMOFP13* coding region (Fig. 4a). Haplotypes could be constructed for 1024 of them, showing a certain geographical distribution of the haplotypes (Fig. 4b). Haplotype A was characteristic of the China and Far East regions (what overlaps with the distribution of ssp. *agrestis* cultivars). Haplotype B (PS haplotype) was the most common haplotype in the Europe, America, Middle and Near East in regions, overlapping with the distribution of ssp. *melo* cultivars. Haplotype C (CALC haplotype) was characteristic of India and CALC, although was also present in America. Haplotype D had a very low frequency. In general, the haplotypes seemed to be mainly distributed geographically, nicely reflecting the history of melon diversification, with a primary center of diversification in India, a divergent diversification to the Western and Eastern regions and certain admixture due to migrations and breeding (Gonzalo et al. 2019). European cultivars have a highly diverse fruit morphology, and in fact, they have been classified in several horticultural groups based on this diversity (Pitrat 2017). However, a very low haplotypes diversity has been found among them. Therefore, the nucleotide variation at *CMOFP13* coding sequence locus could not be related, at least directly, to the fruit morphology variation in melon germplasm.

To further investigate the possible association between *fsqs8.1* locus variability with FSI, 63 accessions from the COMAV’s germplasm bank representing diverse worldwide melon accessions were evaluated in two different experiments. FSI ranged from 0.8 (round fruit) to 4.5 (highly elongated) in the 2014 and 2018 experiments, respectively (Supplementary Fig. S6). Accessions showing very different FSI among experiments were removed. Accessions were genotyped with SNPs located in the *fsqs8.1* locus, but no SNP was significantly associated with FSI. Eight haplotypes were found. However, B, D, E and G haplotypes presented four or less members, so they were removed for haplotype-based association analysis, remaining A, C, F, PS haplotypes. FSI mean for haplotype C (FSI = 1.04, 1.14 in 2014 and 2018 experiments, respectively) was significantly lower (rounder fruit) than the A and PS haplotypes means (ranging between 1.37 and 1.69). Most accessions with the haplotype C belong to the horticultural class cantalupensis, which characteristically produce round fruit (Pitrat 2017). Therefore, the association was not conclusive, as it could be due to the genetic structure of melon cultivars.

The presence of the deletions observed in the CALC and CALC8-1 genomes was also assessed in the 1,175 re-sequenced accessions. Raw sequence reads of 25 accessions (Supplementary Table S4) showing missing data for variants between chromosome 8 position 26,900,00 and 26,942,000 (Fig. 1) were retrieved and mapped de novo to the CM4.0 melon genome. All mappings showed the same pattern as previously observed with CALC and CALC8-1 (Supplementary Fig. S7). Interestingly, two independent replicates of CALC (MS-884 and MS-1155 in Zhao et al. 2019 coding) and MR-1 (ME-1018, a modern cultivar with CALC in its pedigree, Thomas 1986) showed the CALC8-1 read pattern, confirming the presence of the deletion in that genomic region. Most of the accessions showing the deletion read pattern belonged to India or had an Indian origin in the pedigree (MR-1). These results suggest that the origin of this structural variation occurred in an ancestral Indian accession, and it has not been dispersed with the diversification of melon cultivars, except its introduction into the MR-1 cultivar. Even though we did not have access to seeds of these accessions to evaluate their fruit, the Germplasm Resources Information Network (https://www.ars-grin.gov/) provides fruit evaluation data for nine of them. The FSI of those nine accessions ranged from round to elongated (Table 1), again suggesting the non-direct association of the genomic deletions with the variation in FSI. In fact, CALC, the accession donor of *fsqs8.1* round allele, produces elongated fruit (Diaz et al. 2014).

**Table 1:** Characteristics of accessions showing the large deletion in *fsqs8.1* locus according GRIN-USDA passport data.

a	Country of origin					
	USA	India	Japan	Israel	Turkey	Iran
Number of accessions	1	19	1	1	1	1

b	Fruit shape		
	Round	Oval	Long
Number of accessions	2	3	4

1a indicates the number of accessions that belong from different countries. 1b depicts the number of accessions producing different fruit morphology

### Functional validation of *CmOFP13* in *Arabidopsis thaliana*

**Fig. 5:**
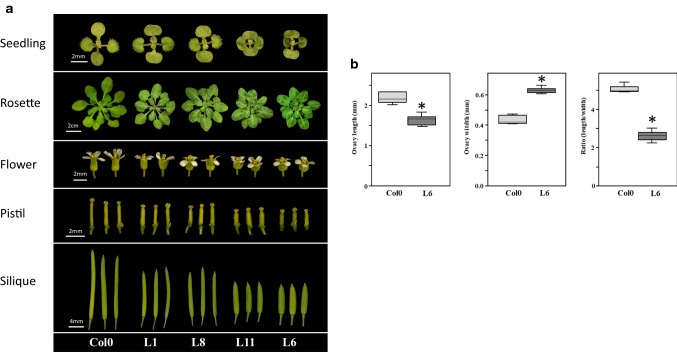
Alteration of organ shape due to overexpression of *CmOFP13* in *Arabidopsis thaliana*. **a** Images of *A*. *thaliana* wild-type plants (Col0) and transgenic plants expressing *CmOFP13* from CALC8-1 showing a mild (L1 and L8) or strong (L6 and L11) phenotype at the 9-day-old seedling stage, adult rosette, flowers, pistils, and mature siliques. Scale bars are depicted in each stage. **b** Comparison between ovary length, width and the length/width ratio between Col-0 and L6 transgenic line. (*) Indicates statistically significant differences (*p* < 0.001)

Thus, to verify whether *CmOFP13* is indeed a functional homolog of *AtOFP1* and *SlOFP20*, playing a role in the control of ovary and fruit shape, we over-expressed the allelic variant from CALC8-1 in *A. thaliana*, under the control of the 35S constitutive promoter from the plant pathogen Cauliflower Mosaic Virus (CaMV). Four transgenic lines were finally selected based on single locus segregation in hygromycin (Fig. 5A). All four lines showed a similar phenotype, although they had differences in penetration, with two mild (L1 and L8) and two strong (L6 and L11) phenotypes. All transgenic seedlings showed cotyledons with shorter petioles, and a protuberant proximal blade, which modified the normal oval shape of the Col0 accession to a kidney-shaped cotyledon. This phenotype continued during development, with round and curled leaves, resulting in compact plant rosettes. More importantly, the overexpression of *CmOFP13* provoked a shortening of flower whorls, including pistils that resulted in shorter fruits. Pistil morphology was measured in the strong L6 line (Fig. 5b). Pistil length was reduced to 75%, whereas pistil width was increased by 43% of the Col-0 wild type. These changes resulted in a 50% reduction of the ratio length to width. Overall, these phenotypes strongly resembled those described upon overexpression of Class 1 OVATE genes from *A. thaliana* and tomato (Hackbusch et al. 2005; Wang et al. 2007, 2011; Wu et al. 2018). Therefore, a higher expression of *CmOFP13* caused reduced ovary and fruit length in transgenic *A. thaliana* plants, which in agreement with the relationship found between the variation in expression of endogenous *CmOFP13* and the variation in fruit shape in CALC8-1, PS and SC8-3. We interpreted that *CmOFP13* was the functional homolog of *AtOFP1* and *SlOFP20*, controlling ovary elongation in melon.

### Genetic effects of *fsqs8.1*

**Fig. 6:**
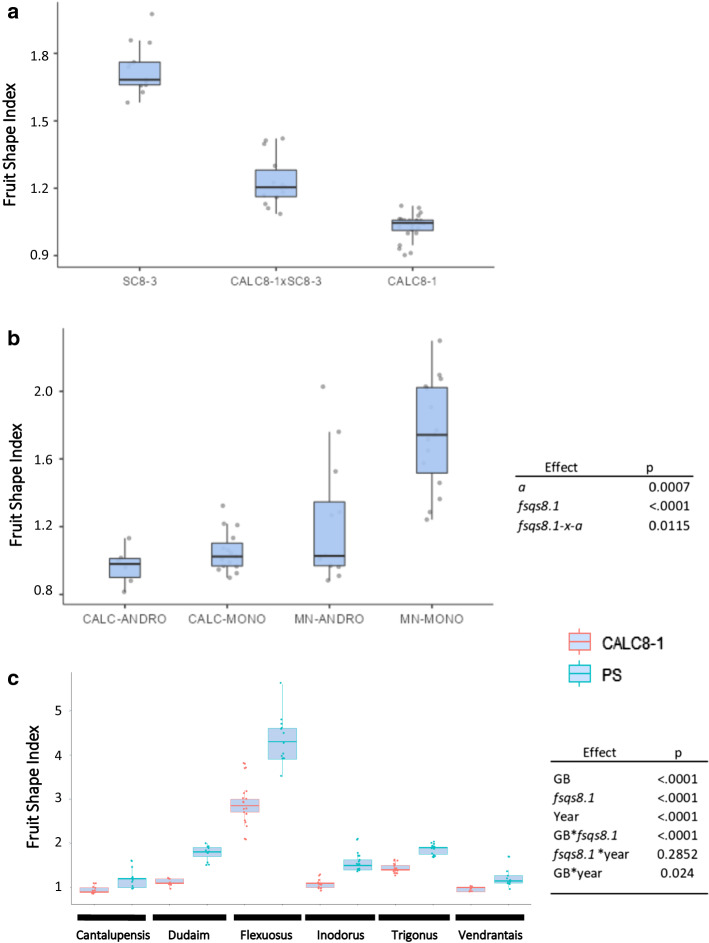
Interactions of *fsqs8.1* in different genetic situations. **a** Comparison of CALC8-1, SC8-3 and CALC8-1-x-SC8-3 hybrid fruit shape indexes. The blue bar indicates the expected mid-parent mean, and the asterisk the significance of the contrast (*p* = 0.0002). **b** Interaction between *a* and *fsqs8.1* in an F_2_ population from the cross between CALC8-1 and ‘Mucha Nesvi’ (MN, a monoecious traditional cultivar). The means for the four homozygous allele combinations for the two loci (CALC/MN) for *fsqs8.1*, ANDRO/MONO for andromonoecious/monoecious (*a*)*,* are shown on the left and the significance of the gene effects and interactions according two-way ANOVA on the right. **c** The fruit shape index of fruits from hybrids between accessions belonging to different melon horticultural groups with PS and CALC8-1 is depicted on the left. On the right, the significance of the effects (GB, genetic background, *fsqs8.1* PS or CALC8-1 allele at the locus, and the two years) and their interactions from the three-way ANOVA, are shown

Three different types of allelic interactions for *fsqs8.*1 were investigated: dominance between CALC and SC alleles, epistatic interaction with the *a* gene and interaction with different genetic background. For the dominance analysis, CALC and SC alleles were confronted in the hybrid CALC8-1-x-SC8-3. The gene action for FSI (d/[a]) was 0.43, and the contrast of FSI means between the hybrid and the expected mid-value from SC8-3 and CALC8-1 was statistically significant, with the hybrid being rounder than expected (*p* = 0.002, Fig. 6a). Therefore, the CALC allele had a partial dominant interaction over the SC allele.

We next studied the epistatic interaction between the *a* gene, which controls sex expression (Boualem et al. 2008) and whether it had pleiotropic effects on FSI (Perin et al. 2002) with *fsqs8.1* (Diaz et al. 2014). The interaction was evaluated in an F_2_ between CALC8-1 and ‘Mucha Nesvi’ (MN), a monoecious ameri cultivar that produces elongated fruit. Homozygous plants for the four genotypic combinations at these two loci were selected at seedling stage with AluCAPS (Boualamen et al. 2008) and *fsqs8.1* SNPs. The effects of both genes on FSI were highly significant, with CALC *fsqs8.1* allele inducing a round fruit and, and the MN *a* allele elongated ones (Fig. 6b). On the other hand, the interaction *fsqs8.1-x-a* was statistically significant, confirming the previous results by Diaz et al. (2014). All the plants homozygous for the CALC *fsqs8.1* allele produced round fruit independently of their sex expression, whereas the effect of *a* on FSI was only evident in plants with the MN alleles at *fsqs8.1*.

Finally, the interaction of the *fsqs8.1* alleles with different genetic background was studied by combining CALC8-1 with six melon accessions with contrasting fruit morphology belonging to different horticultural groups (cantalupensis, charentais, dudaim, flexuosus, inodorus and wild melon), and the hybrids were evaluated in two years (Fig. 6c). Hybrids with PS ranged from slightly round (FSI = 1.2) to very elongated (FSI = 4.2). A similar trend was observed in the CALC8-1 hybrids, although in all cases fruit were rounder compared with their respective PS hybrids. The effects of genetic background (GB) and *fsqs8.1* were highly significant confirming the consistency of *fsqs8.1* effects on diverse GBs. The interaction GB*fsqs8.1 was also significant, due to the variability in the magnitude of the effect across GBs, although *fsqs8.1* induced rounder fruit in all cases. The larger effect was observed mostly in hybrids that included accessions that produced elongated fruit. It is likely that there is a limitation of the roundness of the melon fruit, so the effect of *fsqs8.1* may be stronger in GBs that produce elongated melons.

## Discussion

In the current report, we combined high-resolution mapping together with expression and functional analysis to demonstrate that *CmOFP13*, a member of the melon *OVATE* gene family, is the causal gene underlying the melon fruit shape QTL *fsqs8.1*. The discovery of the *OVATE* gene family started with the study of the genetic control of the pear-shaped fruit in the tomato heirloom variety Yellow Pear (Ku et al. 1999). The pear shape of tomatoes was already studied by early tomato geneticists (Hendrick and Booth 1907; Price and Drinkard 1908) demonstrating that the pear shape was controlled by a single gene, which was named *ovate* (Lindstrom 1926, 1927). Liu et al. (2002) identified and cloned the first *OVATE* gene, which did not match with any known proteins at that time, thus the authors suggested the discovery of a new class of regulatory genes involved in plant growth. Further research demonstrated that *OVATE* was a member of a large gene family present in different plant species (Liu et al. 2014, Dangwai and Das 2018). Thus, Monforte et al. (2014) reported 21 OFP members of the OVATE gene family in the melon genome. The large number of members and the co-segregation of their position with fruit shape QTLs led to the hypothesis that OFP members other than *OVATE* could also be involved in the control of organ shape in different species (Rodriguez et al. 2011; Monforte et al. 2014).

Phylogenetic analysis showed that *CmOFP13* is related to tomato *SlOFP14, SlOF17* and *SlOFP20* and the *A. thaliana AtOFP1*, but not to *OVATE* itself. Wu et al. (2018) demonstrated that *SlOFP20* controlled the morphology of tomato fruit and potato tubers. More recently, the peach member *PpOFP1*, also likely homologue to *SlOFP20*, was found to control the flat fruit shape in this crop (Zhou et al. 2020). Evidences of the role of *SlOFP20* homologues in fruit morphology have also been found in cucumber (Colle et al. 2017) and pepper (Tsaballa et al. 2011). Previously, Hackbusch et al. (2005) showed that the overexpression of *AtOFP1* induced shortened siliques in transgenic *A. thaliana* plants. Even though the role of other OFP members on organ morphology cannot be ruled out (Snouffer et al. 2020), these results suggest strongly that the homologues of *SlOFP20* are crucial for the establishment of organ shape in a large number of plant species.

In tomato and peach, the effect of *SlOFP20* and *PpOFP1* is a consequence of changes in gene expression, not changes in protein function (Wu et al. 2018; Zhou et al. 2020). Similarly, no amino acid changes were found between the CALC and PS *CmOFP13* protein sequences. Therefore, the differences in fruit morphology between CALC8-1 and PS are likely caused by the early higher expression of the gene in CALC8-1, compared with PS, resulting in rounder fruit in CALC8-1. The role of gene expression variation in tomato fruit morphology was already pointed out for *fw2.2*, the first cloned fruit morphology QTL (Cong et al. 2002) and it has been observed in subsequent cloned morphology genes and QTLs: *SUN* (Xiao et al. 2008), *lc* (Munos et al. 2011), *fas* (Xu et al. 2015) and *fw3.2* (Chakrabarti et al. 2013), among others. Therefore, *fsqs8.1* is another example of this conserved mechanism that controls organ morphology.

The *fsqs8.1* locus showed an important structural variation, with two deletions surrounding *CmOFP13*. The deletions were confirmed in the re-sequencings of CALC8-1, two independent CALCs (the donor of *fsqs8.1* introgression in CALC8-1), and a small set of melon accession genomes. This structural variation may have affected regulatory sequences, which could explain the changes in the *CmOFP13* expression patterns observed between genotypes in the current report. In tomato, hundreds of structural variations have been associated to changes in gene expression patterns (Alonge et al. 2020). Concomitantly, in the same species, Eshed and Lippman (2019) pointed out that mutations in 5’ and 3’ cis regulatory elements can alter gene expression, resulting in alleles with quantitative effects. In fact, both *SlOFP20* and *PpOFP1* genomic regions present structural variation (Wu et al. 2018, Zhou et al. 2020). In the case of *fsqs8.1*, the larger deletion region is rich in transposon-related sequences in the reference genome, thus it is likely that transposon activity has been relatively frequent in this region, and that their activity could have been responsible for the observed structural variation. The impact of transposons on the generation of phenotypic diversity underlies the maize domestication gene *tb1* (Zhou et al. 2011). Insertions of retrotransposons in upstream regions of *tb1* causes a differential expression that is responsible for the typical single branching of maize cultivars in contrast with the multiple branching of the wild ancestor teosinte. Recently, the role of transposon activity has garnered more attention. Domínguez et al. (2020) found associations of transposon element insertion polymorphisms with major agronomic traits and secondary metabolites in cultivated tomato. Similarly, evidence of the role of retrotransposons on the evolution of the genome and modification of gene expression has also been reported in melon (Demirci et al. 2021; Sanseverino et al. 2015; Yano et al. 2020). Thus, *fsqs8.1* is perhaps another case of gene expression changes due to structural variation mediated by transposon activity.

Strikingly, the *CmOFP13* coding sequence showed a very low variation across melon germplasm, with all mutations being silent, what suggested that the functionality of this protein is needed for proper melon development and only changes in expression patterns might be allowed. Attempts at finding an association between haplotypes in *fsqs8.1* regions and fruit shape did not provide conclusive results. Previously, Gonzalo and van der Knaap (2008) reported that the effect of *OVATE* was dependent on the genetic background in tomato. Rodriguez et al. (2013) showed that *SlOFP20* was a suppressor of *OVATE,* and that specific allele combinations between *SlOFP20* and *OVATE* should coincide to produce the typical ovate elongated fruit. Moreover, Wu et al. (2018) demonstrated that *slTRM5* (a member of *TONNEAU1 Recruiting Motif* family interacts with *SlOFP20* and can modulate the effects on tomato fruit shape. In fact, Gur et al. (2017) found association between melon fruit shape and SNPs located in the *fsqs8.1* region using a different GWAS panel. Therefore, the effects of OFP genes may depend on the genetic background, and the fact that the OFP family includes a large number of members may cause a buffering effect, thereby limiting the effects of a single OFP gene that would be apparent only in certain allelic combinations. Based on the deletion pattern on the *fsqs8.1* locus, the CALC allele is present with low frequency in Indian germplasm, but it was not associated with fruit morphology in that germplasm. The CALC (and other accessions) genome may contain one or several suppressors of *CmOFP13*, or another gene/genes needed are not expressed at the proper time, resulting in elongated fruit. However, the introduction of the CALC *CmOFP13* allele into the PS genetic background induced the development of round fruit, suggesting that the PS genome lacks the putative suppressors or the other needed gene/genes would be expressed at the proper time. Similarly, the effects of the CALC *CmOFP13* allele on fruit shape were consistent when confronted with different genetic background. These experiments were also carried out in the hybrid PS-x-accessions genetic backgrounds (the genetic background of CALC8-1 is PS), so the putative suppressors might be suppressed or the other needed gene/genes would be expressed, and as consequence CALC *CmOFP13* allele could induce its effects on fruit morphology.

An interesting practical breeding results from this work is the repression of fruit elongation induced by *a* by *CmOFP13*. The development of monoecious cultivars producing round fruits (characteristic of the common market class cantalupensis, galia and honeydew) is desirable, as it eliminates the need for emasculation for crossing make crosses to produce hybrid seeds. However, the effects of the *a* gene on fruit elongation makes difficult the combination of both properties. Therefore, *CmOFP13* round alleles could be introduced in breeding programs to generate such cultivars.

In conclusion, we have demonstrated that the gene underlying the fruit shape QTL *fsqs8.1* is *CmOFP13*, a member of OFP gene family, a likely homologue to the tomato *SlOFP20* gene. The effects of *CmOFP13* in ovary and fruit shape are most probably due to changes in its expression pattern, likely due to a structural variation mediated by transposon activity in the surrounding genomic region, and perhaps by promoting cell elongation by microtubule orientation (Zhang et al. 2020). The effects of *CmOFP13* depend on the genetic background, being evident in the PS background, but not in the original Indian background. Even though no associations were found between SNPs or structural variation in the *fsqs8.1* locus with fruit morphology in melon germplasm in this work, *fsqs8.1* genomic region has been associated in fruit morphology in a wide range of melon germplasm (Monforte et al. 2014; Gur et al. 2017; Pan et al. 2020), actually the region is considered to include a consensus FSI QTL (Monforte et al. 2014; Pan et al. 2020). Other mutations should be involved in those alleles, likely regulatory mutations rather than coding mutations, or additional linked genes. Therefore, the CALC *CmOFP13* allele is a cryptic variant that may have arisen by a structural variation, with a strong potential to become a new breeding tool for generating monoecious cultivars that produce round fruit.

## Supplementary Information

Below is the link to the electronic supplementary material.

Supplementary file1 (PDF 164 KB)

Supplementary file2 (PDF 1785 KB)

## Data Availability

Genome sequences from PS and CALC8-1 are available at NCBI repository BioProject ID PRJNA729905.
