# Supplementary material for: A cryptic variation in a member of the Ovate Family Proteins is underlying the melon fruit shape QTL fsqs8.1
Source: Theor Appl Genet. 2021 Nov 25;135(3):785–801. doi: 10.1007/s00122-021-03998-6 (PMC8942903; doi:10.1007/s00122-021-03998-6)
Supplement: Supplementary file 1 — Supplementary file1 (PDF 164 KB) [file 122_2021_3998_MOESM1_ESM.pdf]

Table S1. Accesions used in different experiments. The first column indicates the accession code, followed by the horticultural group according (Pitrat 2017), the accession code and/or cultivar name, the country of origin and the experiment where they were used

Code	Horticultural group	Accession/Cultivar Name	Origin	Association	GB experiment	Interaction fsqs8.1-x-a
Con-Pat81Ko	Makuwa	Pat 81	Korea	x		
Con-FreeCJa	Conomon	Freeman's Cucumber	Japan	x		
Con-NanChi	Makuwa	Nanbukin	China	x		
Con-ShiroJa	Conomon	Shiro Uri Okayama	Japan	x		
Con-XiaoChi	Makuwa	Xiaobai	China	x		
Can-HBJUSA	Cantalupensis	Ar Hale's Best Jumbo	USA	x		
Can-Nica	Cantalupensis	Nicaragua77	Nicaragua	x		
Can-PearlJa	Cantalupensis	PI 266947, Pearl	Japan	x		
Can-KhLib	Cantalupensis	CUM 294, Khiar	Libya	x		
Con-Chi51Chi	Cantalupensis	China51	China	x		
La-DOgenEur	Cantalupensis	Dvash Ha Ogen	Europe	x		
Can-NOFran	Cantalupensis		France	x		
Can-NYIsr	Cantalupensis	Noy Yizre'el	Israel	x		
La-OgenBul	Cantalupensis	Ogen 1	Bulgaria	x		
Can-PGRFran	Cantalupensis	Petit Gris de Rennes	France	x		
Can-PMRUSA	Cantalupensis	PMR45	USA	x		
Can-PresFran	Cantalupensis	Prescott Fond Blanc	France	x	X	
Can-VedFran	Cantalupensis	Vedrantais	France	x	X	
Can-WMRUSA	Cantalupensis	WMR 29	USA	x		
Can-EAnHun	Cantalupensis	CUM 305, Ezüst Ananasz	Hungary	x		
Am-ChandAfg	Chandalak	PI 276660, VIR 610 Chandalak	Afghanistan	x		
Am-KizilUzb	Ameri	BGV001632, Kizil-uruk	Uzbekistan	x		
Am-SarakIran	Agrestis	Sarakhs	Iran	x		
Flex-KhiIrak	Flexuosus	CUM 344, khiar taaruzy	Irak	x		
In-LaCalSp	Ibericus	BGV003971, E-C-1, Calamonte	Spain	x		
La-BanBul		Plovdiver Banane	Bulgaria	x		
Am-NanaGeorg	Ameri	BGV001367, Nanatri	Georgia	x		
In-MusHung	Cassaba	CUM 133, Muscatello	Hungary	x		
Can-355-Irak	Cantalupensis	CUM 355	Irak	x		
In-RoMoch2Sp	Ibericus	BGV10846, J.A 8221, Mochuelo	Spain	x		
In-KirTur	Cassaba	PI 169305, Kirkagac	Turkey	x		
Am-ImlKaz	Chandalak	PI 476342, Imljskaja	Kazakhstan	x		
Can-PSUSA	Cantalupensis	Persian Small Type	USA	x		
La-MalacAlg		PI 222138, Malacara	Algeria	x		
Mom-KhaInd	Momordica	CUM 438, Kharbuja	India	x		
Can-ASLFran	Cantalupensis	ASL_01.1	France	x		
Am-SouiMor	Ameri	Souilah	Morocco	x		
In-LaCocaSp	Ibericus	BGV000463, AN-C-109, melon coca	Spain	x		
In-LaEscrSp	Ibericus	BGV000487, AN-C-133, melon escrito oloroso	Spain	x		
In-BTempSp	Ibericus	BGV000444, AN-C-9, Blanco tempranillo	Sapin	x		
La-MadASp	Ibericus	C-62, Madura amarilla	Spain	x		
In-PsPiñSp	Ibericus	BGV005018, Piel de sapo Piñonet	Spain	x		
In-PsPipaSp	Ibericus	BGV013188, Ch5, Pipa de oro	Spain	x		
In-AsliTun	Ameri	Melon Aslí	Tunisia	x		
In-PsPiñonSp	Ibericus	BGV003686 CM-C-17, Melon piñoncillo	Spain	x		
In-BlisSp	Ibericus	BGV003692, CM-C-23, Melon blanco	Spain	x		
In-RoMoch1Sp	Ibericus	BGV003718, CM-C-50 Mochuelo	Spain	x		
In-TeLVillSp	Ibericus	BGV003721, CM-C-53, Largo de Villaconejos	Spain	x		
In-MelAmaSp	Ibericus	BGC003999, E-C-29, Melona amarilla	Spain	x		
In-PsVPintSp	Ibericus	BGV004009, E-C-39, Verde pinto	Spain	x		
La-ErizoSp	Ibericus	BGV001410, E-C-3, Eriçó mallorquin	Spain	x		
In-ComunSp	Ibericus	BGV010840, J.A 8214, Común	Spain	x		
In-LaBolasSp	Ibericus	BGV010843, J.A 8217, Bolas	Spain	x		
Am-TokTaj	Ameri	Tokash	Tajikistan	x		
Am-SalUkr	Ameri	PI 506459, Salgirskaja	Ukraine	x		
In-LaInvernSp	Inodorus	BGV10746, J.A 8208, Invernizo	Spain	x		
Am-BirUkr	Ameri	Birjucekutskaja	Ukraine	x		
In-BrancPor	Ibericus	Branco de ribadeteja	Portugal	x		
In-HamiChi	Inodorus	Hami	China	x		
Ag-Hum93Sud	Wild	Humaid93	Soudan	x		
Can-NCFran	Cantalupensis	Noir des Carmes	France	x		
Am-OuzUzb	Ameri	Ouzbeque	Ameri, Uzbekistar	x		
Am-YokIs	Ameri	Yokneam	Israel	x		
Popone		PI 193495 popone	Etiopia	x		
In-CucumIta	Inodorus	CUM 372, Cucumarazzo	Italy	x		
In-HCECSp	Ibericus	BGV003973, E-C-3, Hilo carrete	Spain	x		
Trigonus	Wild	Ames 24294	Pakistan		X	
Dud-QAPMGeorg	Dudaim	Queen Anne Pocket	Afghanistan		x	
In-T111PsSp	Ibericus	Piel de Sapo	Spain		x	
Am-NesviGeor	Ameri	Mucha nesvi	Georgia			x

Table S2. List of primers used in the experiments of this work.

Technique	Transcript id /SNP name	annotation	Forward (5´-3´)	Reverse (5´-3´)	Physical position in Chr8 in CM4.0 reference genome	Observations
qRT-PCR	MELO3C025206	*Transcription repressor OFP1*	CGATGCCTACTTCGTCAATTC	TTTGTCAGTTCGGAAATCAGG	26948106-26947851	
KASPAR	sca76-197		GAAGGTGACCAAGTTCATGCTTGGTGCTATTATGAGAGTCGA<br>GAAGGTCGGAGTCAACGGATTTGGTGCTATTATGAGAGTCGC	TTGATATCAAACCATGATGA	26839235	FAM tail (GAAGGTGACCAAGTTCATGCT): PS;<br>HEX tail (GAAGGTCGGAGTCAACGGATT): CAL8.1
	Sca76-184		GAAGGTGACCAAGTTCATGCTGCACCCACCGTATTAAACAAGC<br>GAAGGTCGGAGTCAACGGATTGCACCCACCGTATTAAACAAGT	GAGACGTTGCACCGGTACTTA	26851732	
	Sca76-192		GAAGGTGACCAAGTTCATGCTCAAATTCACGTGTCACCAAAGG<br>GAAGGTCGGAGTCAACGGATTCAAATTCACGTGTCACCAAAGA	ATGGAAGAGCCTTTTTGAGCTT	26843360	
	Sca76-140		GAAGGTGACCAAGTTCATGCTGGCATAAAGAGCACTAGCTTGG<br>GAAGGTCGGAGTCAACGGATTGGCATAAAGAGCACTAGCTTGA	AAGGTCCCCGCTTTAAATCTT	26896907	
	Sca76-155		GAAGGTGACCAAGTTCATGCTGATGATACAGACAAAGGAGGTTGAA<br>GAAGGTCGGAGTCAACGGATTGATGATACAGACAAAGGAGGTTGAG	TTTGATACTCAACATAATCTGGAAAGA	26879328	
	Sca76-84		GAAGGTGACCAAGTTCATGCT CCCATTAATTCCCAAATGACTCT<br>GAAGGTCGGAGTCAACGGATT CCCATTAATTCCCAAATGACTCG	AAGGTGGATCGGAGATGGTG	26959322	
	Sca76-42		GAAGGTCGGAGTCAACGGATTGGTTAACAAGTCAATTATTTG<br>GAAGGTGACCAAGTTCATGCTGGTTAACAAGTCAATTATTTA	GTTTGCCTCCCTAAAGTAACT	26998784	
PCR	Deletion-marker		ACATCCTAACAACACCAAAACCA	AGGGAGAACATTCGATTTTCAACA	26937196	
	sca76-95indel-ov		CGACTCTGCAACTCACACCTA	TTCTCATCGGAAAATGTTTGG	26948384	
HRM	Sca76-197		GGGGGTGATTTGTTGCTTAG	CTTTGCTTACACTGCGACCA	26839235	
	sca76-192		ATGGAAGAGCCTTTTTGAGC	AAAAGTTGAATCCTTTGCATTTTC	26843360	
	sca76-191		TTGATGTATAGGCTAATTGACACGA	TAGGGTTTAGGATCGAAAGATGA	26844243	
	sca76-168		GCGAAGCATGTTACAGCAAG	TCTTTTTCATCGCCCCTTTA	26866973	
	sca76-156		AAAAATTTGTCCTTTCTTTGGACA	TGGGATACCAACAGAGTTTTGA	26879084	
	sca76-155		CCAAAAAGGCTAAATTGCTGA	AAGGACGTTTCACGTTGACC	26879328	
	sca76-140b		TTTTTATTGGGTCTCTTTTGCAC	GTGCAATATTGGATGAAAAGGTG	26896907	
	sca76-137		CACCTCATCAAATTCTTCTTAGGC	ATTCCAAATTGAGGAATTAAAAACA	26898531	
	SNP8		TCCCCACTTCTTTCTAAGCTCT	GGGGACAAACTCTGAAGACCAA	26953528	
	SNP4		TAGGCGCTTTGTTGACGGA	ATCTGAAGAGCAGACCAGAACA	26957724	
	SNP3		GTCTATTGGCAGTGTGTATGTCA	AGCCAAACCACAATCCTTCA	26958547	
	SNP1		GTTCGAGCATGGGCGATTAG	ACGGTGTTGCCATCAAACAA	26958640	
	sca76-84		GCCGGAGTATTCTCAACAGC	CGACAGGGCCAGAGAGAG	26959322	
	sca76-80		TCACATGTTTTCTTACCGTTACC	CCATTCAATTGTAGTGAATAGTGG	26963844	
	Sca76-45		CAAGAAACAAGAACCATCCTCA	CACTCTTGAGGTTAATCAAAAAGGA	26996244	

Table S3. Progeny testing of *fsqs8.1*

Screening	Parent	Progeny	Sca76-197	sca76-192	sca76-191	Sac76-184	sca76-168-F	sca76-156b	sca76-155	sca76-140	sca76-137a	Deletion-marker	snp-OVATE	sca76-95indel-ov	SNP8	SNP4	SNP3	SNP1	sca76-84	sca76-80	Sca76-45
		Position on chromosome 8	#########	#########	#########	#########	#########	#########	#########	#########	#########	#########	#########	#########	#########	#########	#########	#########	#########	#########	#########
2016	15M36-43	16M5	PS	PS	PS	PS	PS	PS	PS	PS	PS	PS	PS	PS	PS	PS	PS	H	H	H	H
	15M36-100	16M6	PS	PS	PS	H	H	H	H	H	H	H	H	H	H	H	H	H	H	H	H
	15M36-52	16M59	H	H	H	H	PS	PS	PS	PS	PS	PS	PS	PS	PS	PS	PS	PS	PS	PS	PS
	13M33-8	16M4	CALC	CALC	CALC	CALC	CALC	CALC	CALC	CALC	CALC	CALC	CALC	CALC	CALC	CALC	CALC	CALC	CALC	H	H
2019	18M17-168	18M91	H	H	H	H	PS	PS	PS	PS	PS	PS	PS	PS	PS	PS	PS	PS	PS	PS	PS
	18M18-3	18M93	CALC	CALC	CALC	CALC	CALC	CALC	CALC	CALC	CALC	CALC	CALC	CALC	CALC	CALC	CALC	H	H	H	H
	18M14-73	18M92	PS	PS	PS	PS	PS	PS	PS	PS	PS	PS	H	H	H	H	H	H	H	H	H

Screening	Parent	Progeny	Fruit shape progeny test: Average FSI CALC allele (+/- sd)	N	Average FSI PS allele (+/- sd)	N	P-value
2016	15M36-43	16M5	1.19±0.1	8	1.27±0.09	8	n.s
	15M36-100	16M6	0.95±0.04	7	1.26±0.04	7	<0.0001
	15M36-52	16M59	1.31±0.08	8	1.33±0.11	5	n.s
	13M33-8	16M4	1.02±0.04	5	1.00±0.1	11	n.s
2019	18M17-168	18M91	1.62±0.13	7	1.42±0.27	9	n.s
	18M18-3	18M93	0.93±0.06	3	1.07±0.10	3	n.s
	18M14-73	18M92	1.01±0.05	5	1.33±0.16	5	0.003

Table S4. Melon accessions showing the large deletion in the *fsqs8.1* locus from Zhao et al (2019) re-sequencing data.

Individual code	Botanical variety	NMGWM#	GRIN#	Name	Horticultural Gr	Categories	Origin	Province/Site
MS-1018	*C. melo L. ssp. agrestis*			MR-1	momordica	Cultivar	United States	
MS-1019	*C. melo L. ssp. agrestis*		PI 124111-2		momordica	Landrace	India	
MS-1138	*C. melo L. ssp. agrestis*			Slk-v-052	acidulus		Srilanka	
MS-1151	*C. melo L. ssp. melo*			Varanasi local	chandalak		India	Uttar pradesh
MS-1155	*C. melo L. ssp. agrestis*		PI 124112		momordica		India	Bihar
MS-1182	*C. melo L. ssp. agrestis*			IC274029	momordica		India	Punjab
MS-644	*C. melo L. ssp. agrestis*	ZTG00528		Baipimuxianlipi	momordica	Cultivar	Japan	
MS-911	*C. melo L. ssp. agrestis*	ZTG01195	PI 614323		agrestis	Cultivar	India	Rajasthan
MS-654	*C. melo L. ssp. melo*	ZTG00892		Xinyintian No.19	ameri	Cultivar	Japan	
MS-733	*C. melo L. ssp. melo*	ZTG00286	PI 169320		chandalak	Landrace	Turkey	Ankara
MS-792	*C. melo L. ssp. melo*	ZTG00939	PI 140774			Landrace	Iran	Khorasan
MS-814	*C. melo L. ssp. melo*	ZTG00171	PI 381768		chandalak	Landrace	India	Rajasthan
MS-820	*C. melo L. ssp. melo*	ZTG00283	PI 164858-2		chandalak		India	
MS-829	*C. melo L. ssp. agrestis*	ZTG00584	PI 381803		momordica	Landrace	India	Rajasthan
MS-830	*C. melo L. ssp. melo*	ZTG00592	PI 179894		cassaba	Landrace	India	Gujarat
MS-842	*C. melo L. ssp. agrestis*	ZTG00886	PI 164666		dudaim	Landrace	India	Karnataka
MS-843	*C. melo L. ssp. melo*	ZTG00897	PI 216030		chandalak	Landrace	India	Delhi
MS-844	*C. melo L. ssp. melo*	ZTG00899	PI 236030 2		chandalak	Landrace	India	
MS-847	*C. melo L. ssp. agrestis*	ZTG00965	PI 163219	Kakri Luknowi	flexuosus	Landrace	India	Delhi
MS-849	*C. melo L. ssp. melo*	ZTG00967	PI 164331		dehiscent	Landrace	India	Tamil Nadu
MS-854	*C. melo L. ssp. melo*	ZTG01012	PI 164585		chandalak	Landrace	India	Tamil Nadu
MS-855	*C. melo L. ssp. melo*	ZTG01013	PI 165508			Landrace	India	Uttar Pradesh
MS-858	*C. melo L. ssp. melo*	ZTG01030	PI 164481		ameri	Landrace	India	Rajasthan
MS-875	*C. melo L. ssp. agrestis*	ZTG00793	PI 446928		Honeydew	Landrace	Israel	
MS-884	*C. melo L. ssp. agrestis*	ZTG01347	PI 124112		momordica	Landrace	India	Bihar
