# Supplementary material for: A cryptic variation in a member of the Ovate Family Proteins is underlying the melon fruit shape QTL fsqs8.1
Source: Theor Appl Genet. 2021 Nov 25;135(3):785–801. doi: 10.1007/s00122-021-03998-6 (PMC8942903; doi:10.1007/s00122-021-03998-6)
Supplement: Supplementary file 2 — Supplementary file2 (PDF 1785 KB) [file 122_2021_3998_MOESM2_ESM.pdf]

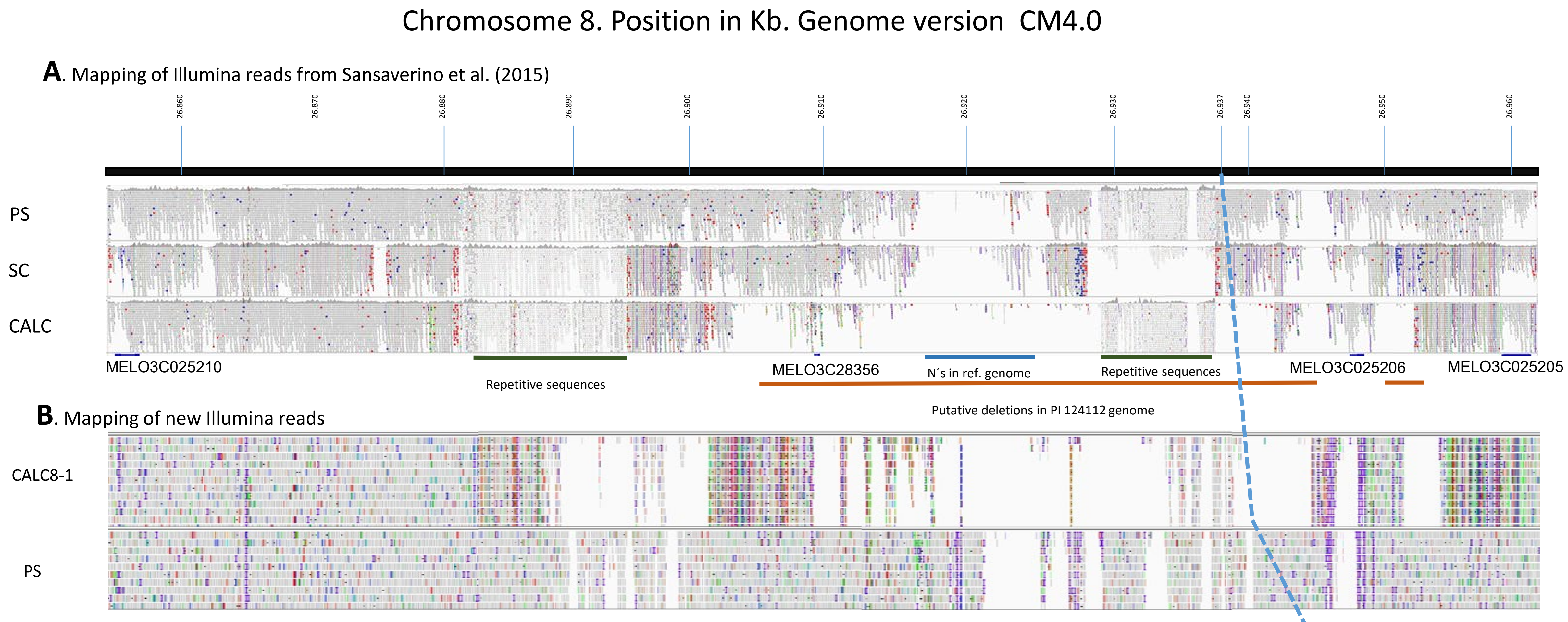

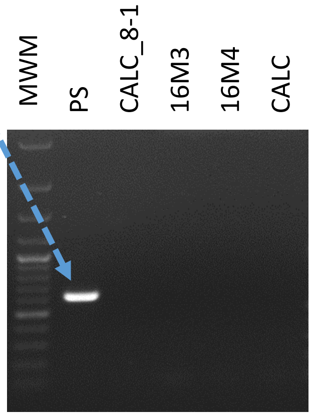

Figure S1. Integrative Genomics Viewer (IGV) snapshots of mapping of Illumina reads in the *fsqs8.1* locus. A) Reads obtained previously by Sanseverino et al. (2015) for PS, PI 161375 (SC) and PI 124112 (CALC). The annotated genes, genome regions with repetitive sequences and, consequently, low quality mapping, a segment with N´s in the reference genome and the extension of the putative deletions are depicted bellow the read mapping. B) Reads obtained in the current research for CALC8-1 and PS. In the lower part, an agarose gel is shown showing the amplification of the deletion marker in PS and lack of amplification in PI 124112 (CALC), the ingrogression line CALC8-1 and recombinant families 16M3 and 16M4 used for the fine mapping (Table S3).

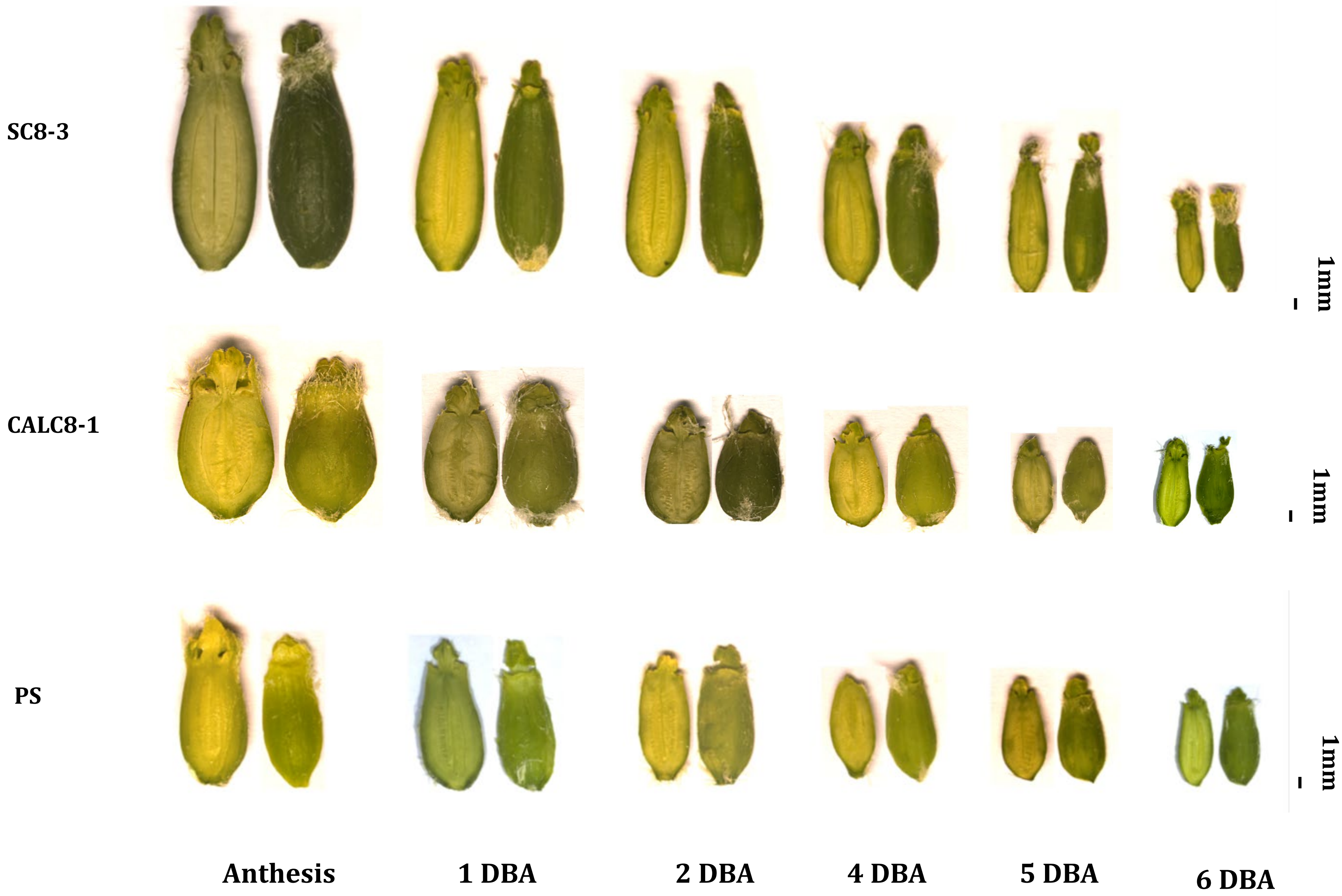

Figure S2. Ovaries of SC8-3, CALC8-1 and PS at anthesis and 1-6 days before-anthesis (DBA)

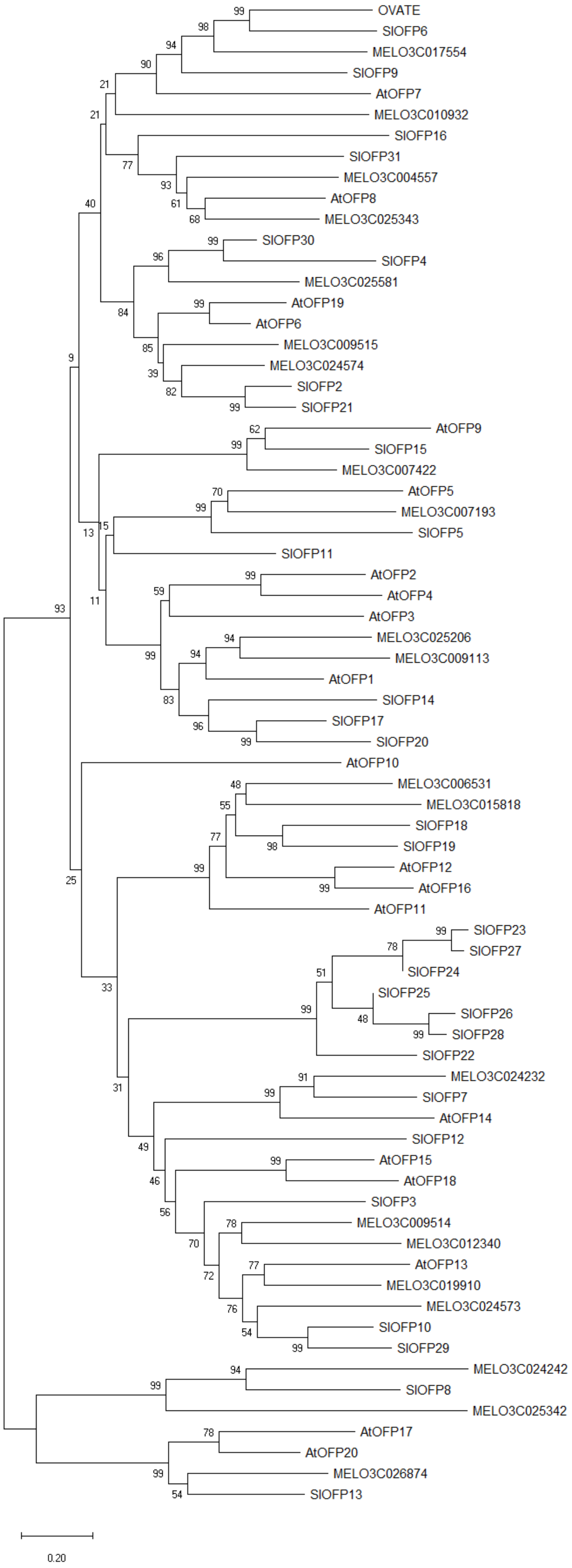

Figure S3. Phylogeny of Arabidopsis thaliana, tomato and melon ovate family protein genes.

Figure S4. Aligment of nucleotide coding and aminoacid sequences of CmOFP13for the introgression lines SC8-3 and CALC8-1, PS and the reference sequence meloc2506

```
T-COFFEE, Version_11.00 Version_11.00
  Cedric Notredame
  CPU TIME:0 sec.
  SCORE=1000
  *
   BAD AVG GOOD
  *
  SC8-3            : 100
  CALC8-1          : 100
  PS               : 100
  melo3c2506_CM4.  : 100
  cons             : 100

  SC8-3            ATGAGAAATCACAAGTTCCGTTTCTCCGACATGATACCCAACGCCTGGTTTTACAAACTCAAA
  CALC8-1          ATGAGAAATCACAAGTTCCGTTTCTCCGACATGATACCCAACGCCTGGTTTTACAAACTCAAA
  PS               ATGAGAAATCACAAGTTCCGTTTCTCCGACATGATACCCAACGCCTGGTTTTACAAACTCAAA
  melo3c2506_CM4.  ATGAGAAATCACAAGTTCCGTTTCTCCGACATGATACCCAACGCCTGGTTTTACAAACTCAAA

  cons             ***************************************************************


  SC8-3            GAAATTGGCGGCGCCTCCAGACCAAAATCTTTCCGTTCCAACAAAAACCCTCACCACCCACCT
  CALC8-1          GAAATTGGCGGCGCCTCCAGACCAAAATCTTTCCGTTCCAACAAAAACCCTCACCACCCACCT
  PS               GAAATTGGCGGCGCCTCCAGACCAAAATCTTTCCGTTCCAACAAAAACCCTCACCACCCACCT
  melo3c2506_CM4.  GAAATTGGCGGCGCCTCCAGACCAAAATCTTTCCGTTCCAACAAAAACCCTCACCACCCACCT

  cons             ***************************************************************


  SC8-3            CCACCTCCCCCGCCCTCCAAACACAAACAACCACCCCCTCCTCCTCCCCACTCTCGTTCCAGA
  CALC8-1          CCACCTCCCCCGCCCTCCAAACACAAACAACCACCCCCTCCTCCTCCCCACTCTCGTTCCAGA
  PS               CCACCTCCCCCGCCCTCCAAACACAAACAACCACCCCCTCCTCCTCCCCACTCTCGTTCCAGA
  melo3c2506_CM4.  CCACCTCCCCCGCCCTCCAAACACAAACAACCACCCCCTCCTCCTCCCCACTCTCGTTCCAGA

  cons             ***************************************************************


  SC8-3            AAATCTTACTATTTCACTAGACAACTCGAATCCAACGATGCCTACTTCGTCAATTCCCCTCCA
  CALC8-1          AAATCTTACTATTTCACTAGACAACTCGAATCCAACGATGCCTACTTCGTCAATTCCCCTCCA
  PS               AAATCTTACTATTTCACTAGACAACTCGAATCCAACGATGCCTACTTCGTCAATTCCCCTCCA
  melo3c2506_CM4.  AAATCTTACTATTTCACTAGACAACTCGAATCCAACGATGCCTACTTCGTCAATTCCCCTCCA

  cons             ***************************************************************


  SC8-3            CCGTCGCCTCCGCTTCTACCGGTACCACTCCCCCCGAGAAAGTCAACAAAACAACTCAAACCA
  CALC8-1          CCGTCGCCTCCGCTTCTACCGGTACCAATCCCCCCGAGAAAGTCAACAAAACAACTCAAACCA
  PS               CCGTCGCCTCCGCTTCTACCGGTACCAATCCCCCCGAGAAAGTCAACAAAACAACTCAAACCA
  melo3c2506_CM4.  CCGTCGCCTCCGCTTCTACCGGTACCAATCCCCCCGAGAAAGTCAACAAAACAACTCAAACCA

  cons             *************************** ***********************************
```

```
SC8-3            GGAAGAAAACAAACGAGTTCCCGGTCCTCCGCCAAGCTCCTCAGCTCCTCCTCCGTCGGCTGC
CALC8-1          GGAAGAAAACAAACGAGTTCCCGGTCCTCCGCCAAGCTCCTCAGCTCCTCCTCCGTCGGCTGC
PS               GGAAGAAAACAAACGAGTTCCCGGTCCTCCGCCAAGCTCCTCAGCTCCTCCTCCGTCGGCTGC
melo3c2506_CM4.  GGAAGAAAACAAACGAGTTCCCGGTCCTCCGCCAAGCTCCTCAGCTCCTCCTCCGTCGGCTGC

cons             ***************************************************************


SC8-3            AGCTGCCACACAACGGCGGAATCTATCTGGACAAAATCCGATTCTCCTCCAGAATTCTCCACT
CALC8-1          AGCTGCCACACAACGGCGGAATCTATCTGGACAAAATCCGATTCTCCTCCAGAATTCTCCACT
PS               AGCTGCCACACAACGGCGGAATCTATCTGGACAAAATCCGATTCTCCTCCAGAATTCTCCACC
melo3c2506_CM4.  AGCTGCCACACAACGGCGGAATCTATCTGGACAAAATCCGATTCTCCTCCAGAATTCTCCACC

cons             ************************************************************** 


SC8-3            TCACCCTCCGACACCTCCCCTGATTTCCGAACTGACAAAATCCTCACTGCCGAAGCATCCAAA
CALC8-1          TCACCCTCCGACACCTCCCCTGATTTCCGAACTGACAAAATCCTCACTGCCGAAGCATCCAAA
PS               TCACCCTCCGACACCTCCCCTGATTTCCGAACTGACAAAATCCTCACTGCCGAAGCATCCAAA
melo3c2506_CM4.  TCACCCTCCGACACCTCCCCTGATTTCCGAACTGACAAAATCCTCACTGCCGAAGCATCCAAA

cons             ***************************************************************


SC8-3            CACTTCGAGCACGACATCGTAATCGATGTATCGTCGAATTACTCCAACAATGCCGTCATCGGC
CALC8-1          CACTTCGAGCACGACATCGTAATCGACGTATCGTCGAATTACTCCAACAATGCCGTCATCGGC
PS               CACTTCGAGCACGACATCGTAATCGACGTATCGTCGAATTACTCCAACAATGCCGTCATCGGC
melo3c2506_CM4.  CACTTCGAGCACGACATCGTAATCGACGTATCGTCGAATTACTCCAACAATGCCGTCATCGGC

cons             ************************** ************************************


SC8-3            GCCTTTGACGAACTGGAACTCCCGCCGATCATCACGAAACAGAGGAAGAAAACAGAGACAAAA
CALC8-1          GCCTTTGACGAACTGGAACTCCCGCCGATCATCACGAAACAGAGGAAGAAAACAGAGACAAAA
PS               GCCTTTGACGAACTGGAACTCCCGCCGATCATCACGAAACAGAGGAAGAAAACAGAGACAAAA
melo3c2506_CM4.  GCCTTTGACGAACTGGAACTCCCGCCGATCATCACGAAACAGAGGAAGAAAACAGAGACAAAA

cons             ***************************************************************


SC8-3            CAGAGAACGACGACGACAACGACGGCAGGAACAAAGAAAGTTGCGGGGAATTCCCCGGGCGTA
CALC8-1          CAGAGAACGACGACGACAACGACGGCAGGAACAAAGAAAGTTGCGGGGAATTCCCCGGGCGTA
PS               CAGAGAACGACGACGACAACGACGGCAGGAACAAAGAAAGTTGCGGGGAATTCCCCGGGCGTA
melo3c2506_CM4.  CAGAGAACGACGACGACAACGACGGCAGGAACAAAGAAAGTTGCGGGGAATTCCCCGGGCGTA

cons             ***************************************************************


SC8-3            CGGCTGCGGATTCACTCCCCGAAAATTGGGTACCGGAAAATGGGAGGGAGGAAAAGCGTATCG
CALC8-1          CGGCTGCGGATTCACTCCCCGAAAATTGGGTACCGGAAAATGGGAGGGAGGAAAAGCGTTTCG
PS               CGGCTGCGGATTCACTCCCCGAAAATTGGGTACCGGAAAATGGGAGGGAGGAAAAGCGTTTCG
melo3c2506_CM4.  CGGCTGCGGATTCACTCCCCGAAAATTGGGTACCGGAAAATGGGAGGGAGGAAAAGCGTTTCG

cons             *********************************************************** ***
```

```
SC8-3               TCGCGGCGGAGCTTGTCGGAGAGTTTAGCGATAATGAAATCATCGTACGATCCACAAAAGGAC
CALC8-1             TCACGGCGGAGCTTGTCGGAGAGTTTAGCGATAATGAAATCATCGTACGATCCACAAAAGGAC
PS                  TCACGGCGGAGCTTGTCGGAGAGTTTAGCGATAATGAAATCATCGTACGATCCACAAAAGGAC
melo3c2506_CM4.     TCACGGCGGAGCTTGTCGGAGAGTTTAGCGATAATGAAATCATCGTACGATCCACAAAAGGAC

cons                ** ************************************************************


SC8-3               TTCAGAAAATCAGTGGGGGAGATGATTGTTGAGAATAACATTAGGGGTTCGAAAGAATTGGAA
CALC8-1             TTCAGAGAATCAATGGTGGAGATGATTGTTGAGAATAACATTAGGGGTTCGAAAGAATTGGAA
PS                  TTCAGAGAATCAATGGTGGAGATGATTGTTGAGAATAACATTAGGGGTTCGAAAGAATTGGAA
melo3c2506_CM4.     TTCAGAGAATCAATGGTGGAGATGATTGTTGAGAATAACATTAGGGGTTCGAAAGAATTGGAA

cons                ****** ***** *** **********************************************


SC8-3               GATCTTCTTGCATGTTATCTGTGTTTGAACGCCGATGAATATCATGATCTTATTATCAAAGTT
CALC8-1             GATCTTCTTGCATGTTATCTGTGTTTGAACGCCGATGAATATCATGATCTTATTATCAAAGTT
PS                  GATCTTCTTGCATGTTATCTGTGTTTGAACGCCGATGAATATCATGATCTTATTATCAAAGTT
melo3c2506_CM4.     GATCTTCTTGCATGTTATCTGTGTTTGAACGCCGATGAATATCATGATCTTATTATCAAAGTT

cons                ***************************************************************


SC8-3               TTTAAGCAGATCTGGTTTGATCTTACGCAACCTTCTCCTCCACCTCTT
CALC8-1             TTTAAGCAGATCTGGTTTGATCTTACGCAACCTTCTCCTCCACCTCTT
PS                  TTTAAGCAGATCTGGTTTGATCTTACGCAACCTTCTCCTCCACCTCTT
melo3c2506_CM4.     TTTAAGCAGATCTGGTTTGATCTTACGCAACCTTCTCCTCCACCTCTT

cons                ************************************************
```

Figure S4. Alignment of CALC8-1, PS and SC8-3 DNA coding and protein amino acid Melo3C020526 sequences

```
T-COFFEE, Version_11.00 Version_11.00
  Cedric Notredame
  CPU TIME:0 sec.
  SCORE=1000
  *
   BAD AVG GOOD
  *
  SC8-3       : 100
  CALC8-1     : 100
  PS          : 100
  melo3c2506  : 100
  cons        : 100

  SC8-3       MRNHKFRFSDMIPNAWFYKLKEIGGASRPKSFRSNKNPHHPPPPPPPSKHKQPPPPPPHSRSRKSYYFT
  CALC8-1     MRNHKFRFSDMIPNAWFYKLKEIGGASRPKSFRSNKNPHHPPPPPPPSKHKQPPPPPPHSRSRKSYYFT
  PS          MRNHKFRFSDMIPNAWFYKLKEIGGASRPKSFRSNKNPHHPPPPPPPSKHKQPPPPPPHSRSRKSYYFT
  melo3c2506  MRNHKFRFSDMIPNAWFYKLKEIGGASRPKSFRSNKNPHHPPPPPPPSKHKQPPPPPPHSRSRKSYYFT

  cons        *********************************************************************


  SC8-3       RQLESNDAYFVNSPPPSPPLLPVPLPPRKSTKQLKPGRKQTSSRSSAKLLSSSSVGCSCHTTAESIWTK
  CALC8-1     RQLESNDAYFVNSPPPSPPLLPVPIPPRKSTKQLKPGRKQTSSRSSAKLLSSSSVGCSCHTTAESIWTK
  PS          RQLESNDAYFVNSPPPSPPLLPVPIPPRKSTKQLKPGRKQTSSRSSAKLLSSSSVGCSCHTTAESIWTK
  melo3c2506  RQLESNDAYFVNSPPPSPPLLPVPIPPRKSTKQLKPGRKQTSSRSSAKLLSSSSVGCSCHTTAESIWTK

  cons        ************************:********************************************


  SC8-3       SDSPPEFSTSPSDTSPDFRTDKILTAEASKHFEHDIVIDVSSNYSNNAVIGAFDELELPPIITKQRKKT
  CALC8-1     SDSPPEFSTSPSDTSPDFRTDKILTAEASKHFEHDIVIDVSSNYSNNAVIGAFDELELPPIITKQRKKT
  PS          SDSPPEFSTSPSDTSPDFRTDKILTAEASKHFEHDIVIDVSSNYSNNAVIGAFDELELPPIITKQRKKT
  melo3c2506  SDSPPEFSTSPSDTSPDFRTDKILTAEASKHFEHDIVIDVSSNYSNNAVIGAFDELELPPIITKQRKKT

  cons        *********************************************************************


  SC8-3       ETKQRTTTTTTAGTKKVAGNSPGVRLRIHSPKIGYRKMGGRKSVSSRRSLSESLAIMKSSYDPQKDFRK
  CALC8-1     ETKQRTTTTTTAGTKKVAGNSPGVRLRIHSPKIGYRKMGGRKSVSSRRSLSESLAIMKSSYDPQKDFRE
  PS          ETKQRTTTTTTAGTKKVAGNSPGVRLRIHSPKIGYRKMGGRKSVSSRRSLSESLAIMKSSYDPQKDFRE
  melo3c2506  ETKQRTTTTTTAGTKKVAGNSPGVRLRIHSPKIGYRKMGGRKSVSSRRSLSESLAIMKSSYDPQKDFRE

  cons        ********************************************************************:


  SC8-3       SVGEMIVENNIRGSKELEDLLACYLCLNADEYHDLIIKVFKQIWFDLTQPSPPPL
  CALC8-1     SMVEMIVENNIRGSKELEDLLACYLCLNADEYHDLIIKVFKQIWFDLTQPSPPPL
  PS          SMVEMIVENNIRGSKELEDLLACYLCLNADEYHDLIIKVFKQIWFDLTQPSPPPL
  melo3c2506  SMVEMIVENNIRGSKELEDLLACYLCLNADEYHDLIIKVFKQIWFDLTQPSPPPL

  cons        *: ****************************************************
```

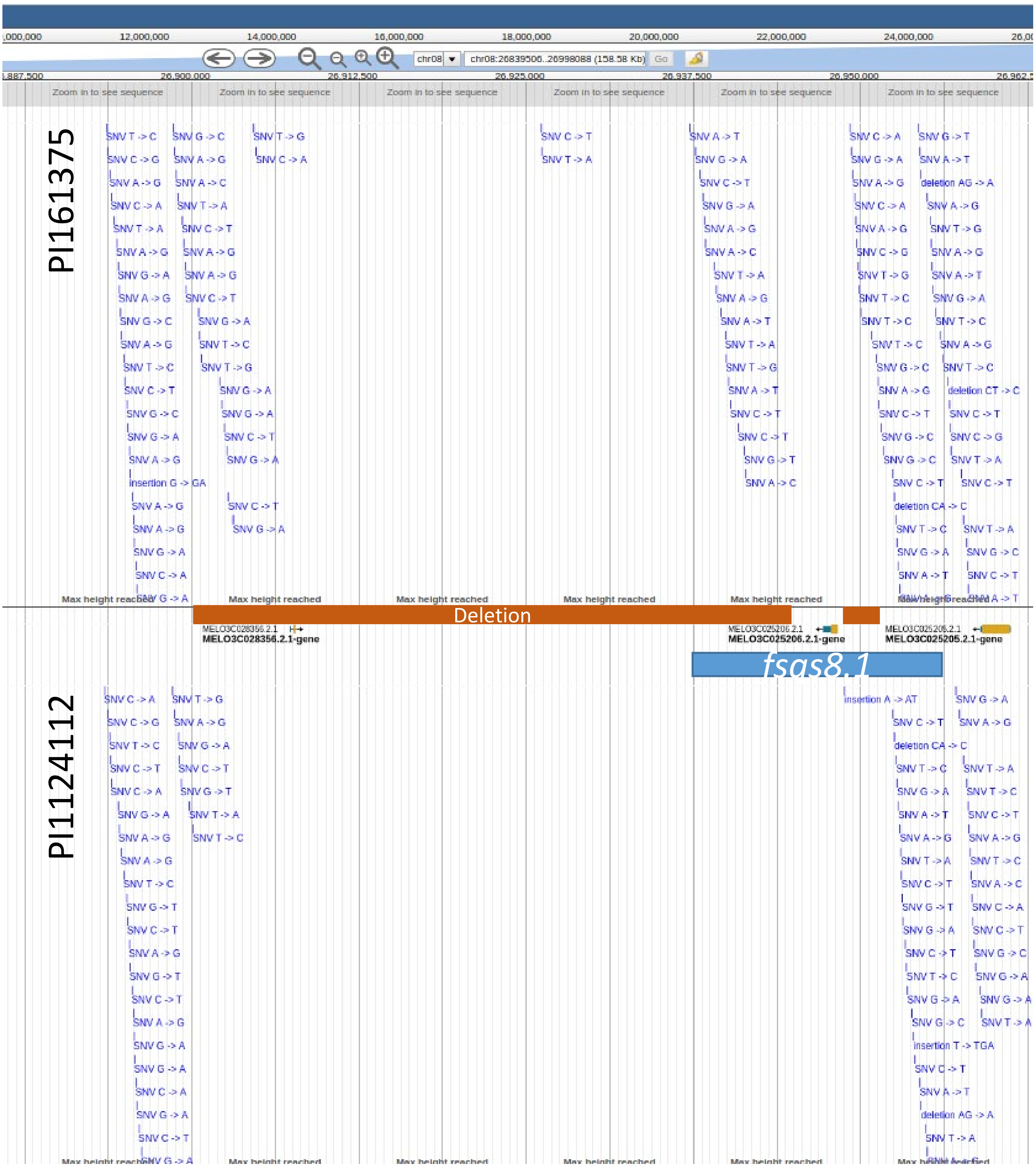

Figure S5. Snapshot of the Jbrowser showing the sequence variations in PI161375 and PI124112 with the reference genome (PS) surrounding the *MELO3C025206* gene. Due to graphic limitations only one SNP every 50 bp is shown. The extend of the deletions and the *fsqs8.1* locus according the fine mapping is also depicted.

sca76-192	sca76-155	sca76-140	sca76-95indel-ov	Haplotype	2018 Number Accessions	2018 FS	2018 HSD	2014 Number Accessions	2014 FS	2014 HDS
CALC	CALC	CALC	CALC	A	5	1.69±0.73	A	5	1.68±0.72	A
CALC	CALC	CALC	PS	B	2	1.27±0.28		2	1.04±0.27	
CALC	CALC	PS	PS	C	12	1.14±0.21	B	12	1.04±0.21	BC
CALC	PS	PS	CALC	D	4	1.13±0.2		4	1.13±0.21	
PS	CALC	PS	PS	E	2	2.66±1.13		2	2.07±0.57	
PS	PS	PS	CALC	F	7	1.21±0.28	AB	6	1.13±0.26	AB
CALC	CALC	PS	CALC	G	4	1.13±0.11		4	1.09±0.1	
PS	PS	PS	PS	PS	26	1.46±0.3	A	26	1.37±0.30	A

Figure S6. Haplotypes in the fsqs8.1 locus in the COMAV germplasm collection and their association to fruit shape.

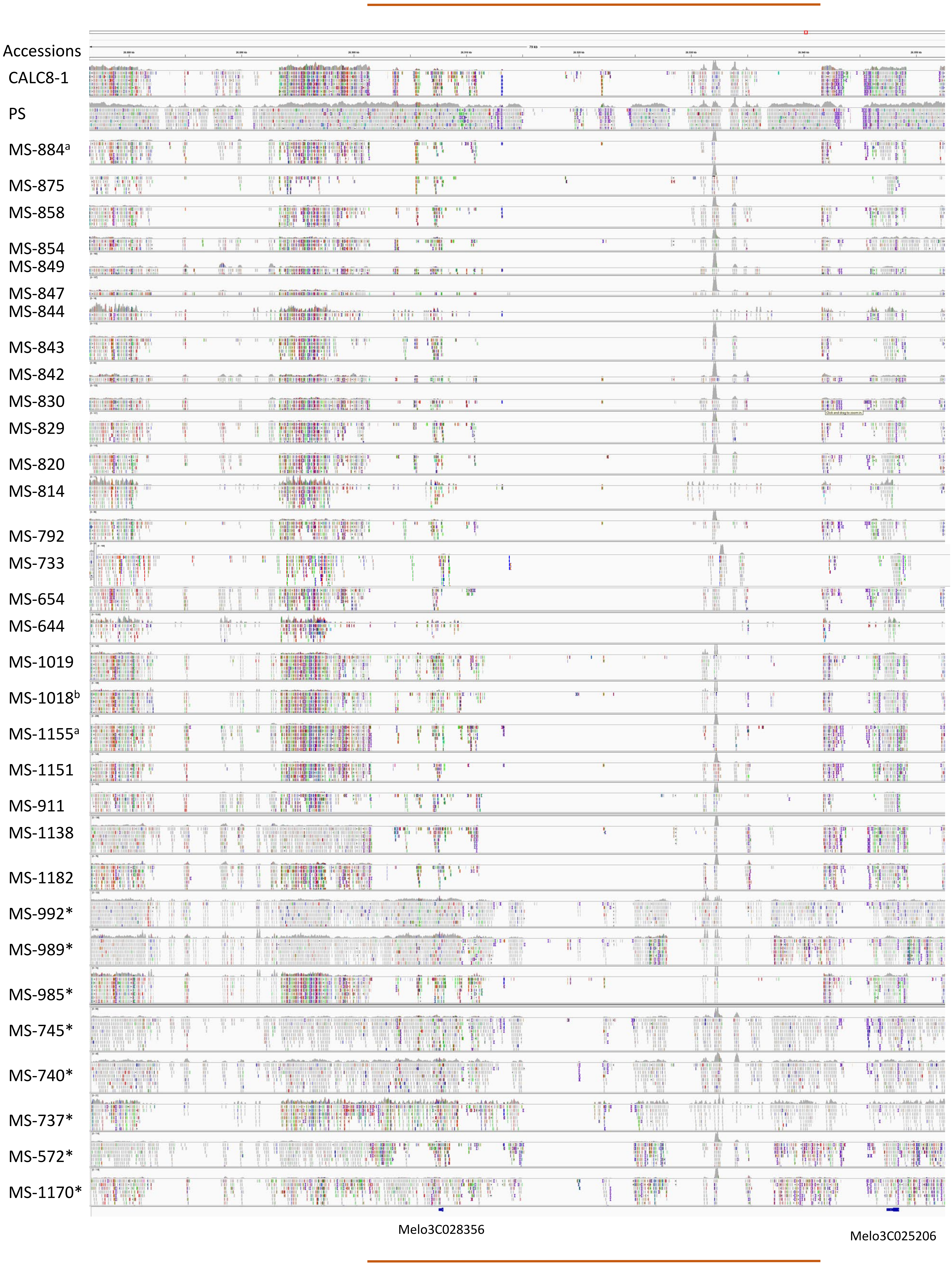

Figure S7. Mapping of Illumina reads from Zhao et al. (2019) re-sequencing data displayed by the Integrative Genomics Viewer. The accessions marked with (*) correspond with randomly selected accessions that did not show the deletion pattern in the SNP calling, while the rest did show it. (a) Independent PI 124112 (CALC) accessions obtained from different sources, (b) the cultivar MR-1 that was developed from CALC.
